# NF‐κB signaling in inflammation and cancer

**DOI:** 10.1002/mco2.104

**Published:** 2021-12-16

**Authors:** Tao Zhang, Chao Ma, Zhiqiang Zhang, Huiyuan Zhang, Hongbo Hu

**Affiliations:** ^1^ Cancer Center and Center for Immunology and Hematology West China Hospital Sichuan University Chengdu Sichuan China; ^2^ Immunobiology and Transplant Science Center Houston Methodist Hospital Houston Texas USA

**Keywords:** cancer, immunity, inflammation, NF‐κB, signal transduction

## Abstract

Since nuclear factor of κ‐light chain of enhancer‐activated B cells (NF‐κB) was discovered in 1986, extraordinary efforts have been made to understand the function and regulating mechanism of NF‐κB for 35 years, which lead to significant progress. Meanwhile, the molecular mechanisms regulating NF‐κB activation have also been illuminated, the cascades of signaling events leading to NF‐κB activity and key components of the NF‐κB pathway are also identified. It has been suggested NF‐κB plays an important role in human diseases, especially inflammation‐related diseases. These studies make the NF‐κB an attractive target for disease treatment. This review aims to summarize the knowledge of the family members of NF‐κB, as well as the basic mechanisms of NF‐κB signaling pathway activation. We will also review the effects of dysregulated NF‐κB on inflammation, tumorigenesis, and tumor microenvironment. The progression of the translational study and drug development targeting NF‐κB for inflammatory diseases and cancer treatment and the potential obstacles will be discussed. Further investigations on the precise functions of NF‐κB in the physiological and pathological settings and underlying mechanisms are in the urgent need to develop drugs targeting NF‐κB for inflammatory diseases and cancer treatment, with minimal side effects.

## Graphical abstract

This year (2021) marks the 35th anniversary of the discovery of NF‐κB. With so many years of in‐depth research on NF‐κB, people have realized that the NF‐κB signaling pathway plays an important role in inflammation, immunity, cell survival and proliferation. This review summarizes the relevant knowledge of the NF‐κB signaling pathway, inflammation, and cancer. The progression of the translational study and drug development targeting NF‐κB for inflammatory diseases and cancer treatment and the potential obstacles will also be discussed.

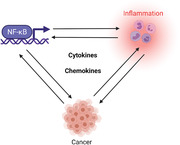

## 1 INTRODUCTION

A nuclear transcription factor is a type of protein that binds to a sequence of conserved nucleotides in the gene's promoter region to initiate the transcription of this gene. The nuclear factor of κ‐light chain of enhancer‐activated B cells (NF‐κB) is one of the nuclear transcription factors with an extensive range of biological functions, which exists in almost all types of mammal cells. In 1986, Sen and Baltimore discovered NF‐κB for the first time through its interaction with a defined site in the enhancer of the κ chain of immunoglobulin gene in B cells.^1^ Since then, intensive studies have established the critical roles of NF‐κB on diverse biological processes, including cell proliferation, metastasis, response to DNA damage, apoptosis, and immune response through its vast target genes.^2^, ^3^ Therefore, NF‐κB is tightly correlated with human diseases such as inflammation, cancer, and autoimmune diseases,^4^, ^5^ and understanding the regulating mechanism and function of the NF‐κB signaling pathway is not only important for revealing the fundamental principles of cellular biology but also crucial to study the pathogenesis and treatment of many human diseases. In this review, we will summarize the progress of NF‐κB study focusing on the crucial roles of this transcriptional factor family in many aspects of inflammation and cancers in the last 35 years, highlighting NF‐κB as a potential therapeutic target for human inflammatory disease and cancer treatment.

## 2 THE NF‐ΚB PATHWAY

### 2.1 NF‐κB/Rel protein family

**FIGURE 1:**
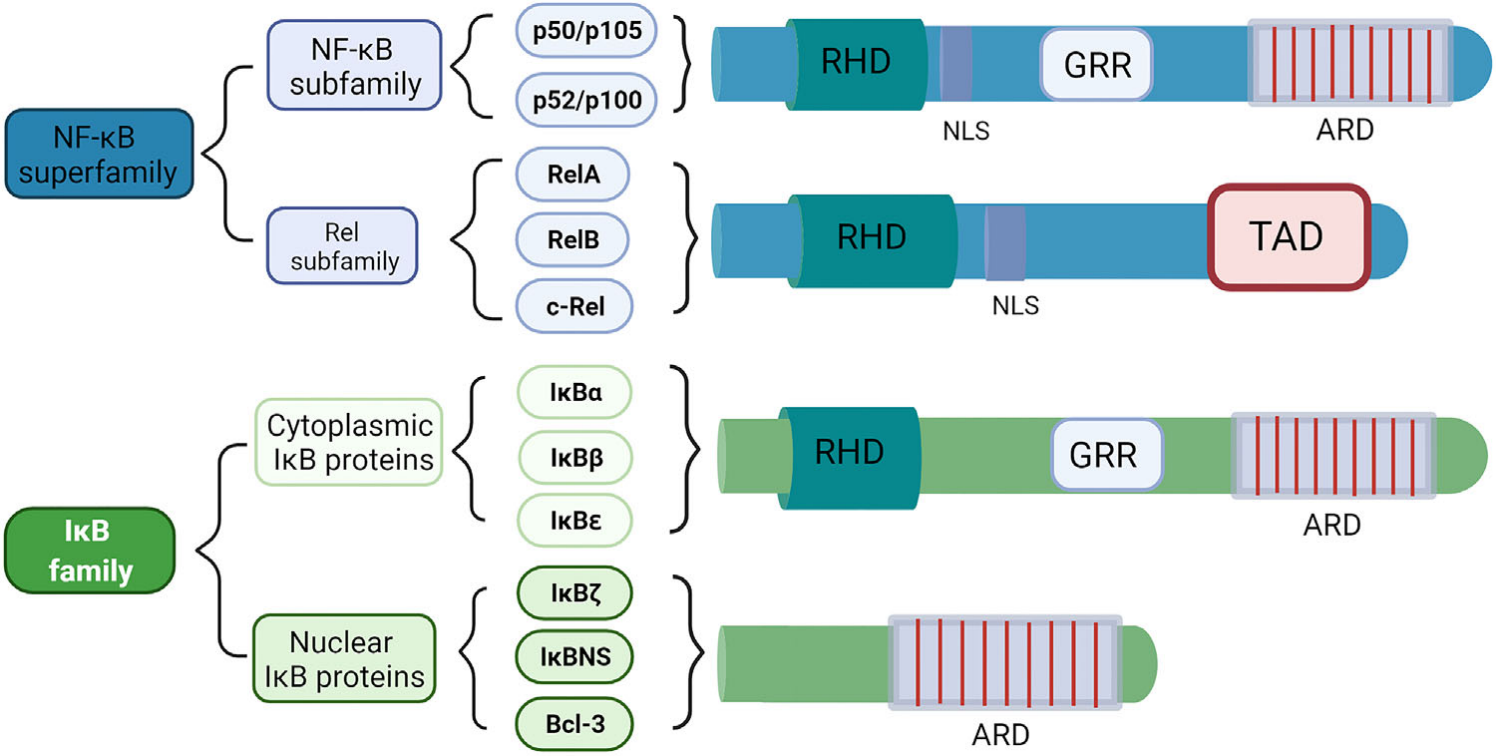
Schematic structures of nuclear factor of κ‐light chain of enhancer‐activated B cells (NF‐κB) superfamily and the inhibitor of NF‐κB (IκB) family. The schematic structures of NF‐κB subfamily proteins, Rel subfamily proteins, and IκB family proteins (cytoplasmic IκB proteins and nuclear IκB proteins) are shown. ARD, ankyrin repeat domain; GRR, glycine‐rich region; NLS, nuclear localization sequence; RHD, Rel homology domain; TAD, transactivation domain

In humans, the NF‐κB superfamily comprises five transcription factors: NF‐κB1 (p50), NF‐κB2 (p52), RelA (p65), RelB, and REL (c‐Rel).^6^ These proteins all contain a Rel homology domain (RHD), a conserved N‐terminal domain, which is very important for DNA binding, dimerization, and nuclear localization. According to the similarity of amino acid sequence within the RHD in the C‐terminal, the NF‐κB superfamily is generally divided into two subfamilies: NF‐κB subfamily (p50 and p52) and Rel subfamily (Rel, RelA, and RelB). NF‐κB1 (p50) and NF‐κB2 (p52) are produced by the processing of protein precursor, p105 and p100, respectively, with ankyrin (ANK) repeat domains in the C‐terminal, which form dimers with other NF‐κB family members. The inhibitor of the NF‐κB (IκB) protein family controls the activation process of NF‐κB by interacting with transcriptional factors. The proteins bind and persist in the cytoplasm, while the Rel protein (Rel, RelA, RelB) has no protein precursor but a conserved transactivation domain in the C‐terminal.^7^ These members of the NF‐κB family share a highly conserved 3000‐amino‐acid RHD, which plays a crucial role in their function.^8^, ^9^, ^10^ In most cases, all five NF‐κB proteins could form homodimers and heterodimers with each other to start gene transcription.^11^ However, RelB is a special exception, which only forms heterodimers. The most common NF‐κB heterodimer is mainly composed of NF‐κB1 (p50) and RelA (p65) (Figure 1). Whether NF‐κB dimer activates transcription or inhibits transcription depends on the DNA regions it binds to and the interaction with other transcription factors.^12^, ^13^

### 2.2 The IκB protein family

The activity of NF‐κB dimer is regulated by the IκB protein. The IκB family is characterized by ANK repeats that interact with the RHD domain of NF‐κB protein.^9^ The IκB protein family has eight members including IκBα, IκBβ, IκBɛ, IκBζ, IκBNS, Bcl‐3, p100, and p105, and every member contains a series of ANK repeat domain (ARD)^14^ (Figure 1). The ARD of IκBs forms a slightly curved cylindrical structure, which contains amino acid residues that specifically recognize and bind to NF‐κB dimers. These repetitive sequences can interact with RHD, allowing IκB to bind to NF‐κB, preventing NF‐κB from entering the nucleus.^11^, ^15^, ^16^ The activation of NF‐κB relies on the degradation of IκB protein. Upon activation, IκBα is phosphorylated by IκB kinase (IKK) complex and ubiquitinated and degraded through the proteasome‐dependent pathway, releasing NF‐κB into the nucleus for transcription to activate target genes. NF‐κB interacts with newly synthesized IκB in the nucleus and exits the nucleus into the cytoplasm, forming the cycle of a non‐phosphorylated nuclear‐cytoplasmic shuttling and inactivation.^10^, ^16^, ^17^ It should be noted that the C‐terminal sequence of the precursor protein p100/p105 of p50/p52 also contains the ANK repeat sequence like IκB, which allows p100/p105 to inhibit the activity of NF‐κB like IκB.^18^, ^19^

### 2.3 Activation of NF‐κB

The NF‐κB activation signaling pathways have been well studied. There are two types of NF­κB activation signaling pathways: the canonical and the noncanonical pathways,^20^ relying on distinct molecular mechanisms to activate NF‐κB, as well as the different sets of target genes involved in cell proliferation, differentiation, and immune response.^16^, ^21^

The IKKs complex is a large multi‐component protein kinases complex, including two homologous catalytically active subunits IKKα (IKK1) and IKKβ (IKK2) and an auxiliary subunit IKKγ (NEMO, NF‐kappaB essential modulator).^22^ IKK can be activated by various stimuli such as growth factors, cytokines, stress factors, and microbial components.^17^ The two catalytically active IKK subunits participate in different pathways. The noncanonical NF‐κB activation pathway is mainly regulated by IKKα, while the regulation of the canonical NF‐κB activation pathway induced by pro‐inflammatory cytokines and various microbial products requires the participation of IKKβ. The oligomerization of IKKα/β/γ can affect the activity of IKKβ.^17^, ^23^, ^24^ Although IKKγ has no catalytic activity, it is necessary for the canonical pathway. IKKγ can form an IKK complex with other IKKs through its N‐terminal, and its C‐terminal can mediate the interaction with the upstream signal adaptor.^16^, ^22^, ^25^

**FIGURE 2:**
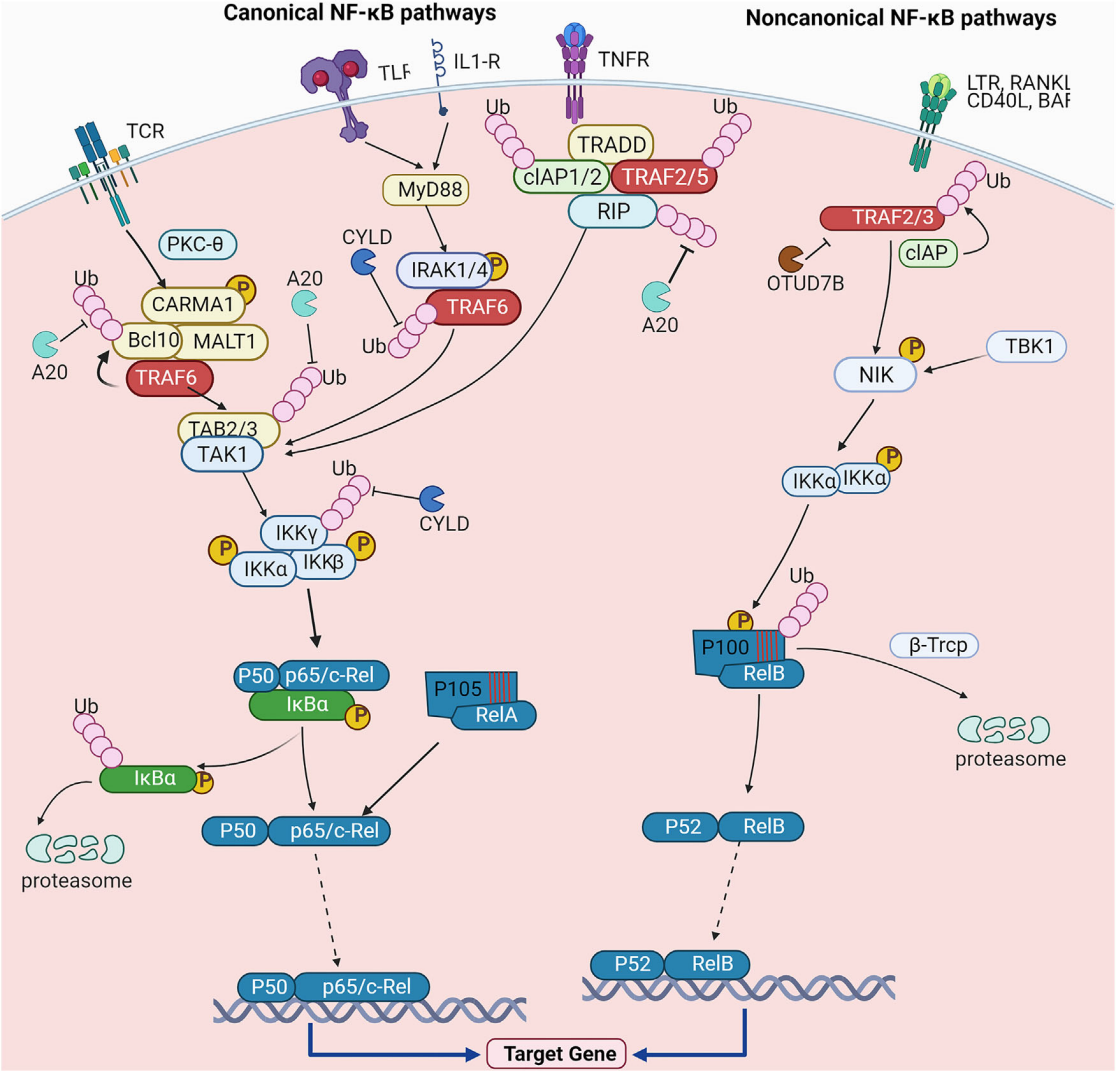
Activation of canonical and noncanonical NF‐κB pathways. In the canonical NF‐κB pathway, protein kinase C‐θ recruits Bcl10 and membrane‐associated lymphoid tissue 1 (MALT1) to form a CARD‐containing MAGUK protein 1 (CARMA1)/Bcl10/membrane‐associated lymphoid tissue (MALT) 1 complex after T‐cell receptor stimulation. TRAF6 is recruited by MALT1, which mediates the ubiquitination of itself and Bcl10 and further activates TAK1. TAK1 phosphorylates and activates IKKβ to induce phosphorylation and degradation of IκBα. After IκBα is degraded, free NF‐κB enter the nucleus to drive target gene transcription. TRADD, the interacting E3 ubiquitin ligases cellular inhibitor of apoptosis (cIAP) 1/2, TRAF2/5, and protein kinase RIP are recruited by tumor necrosis factor receptor (TNFR) stimulation. RIP is then ubiquitinated and recruited to form the TAK1‐IKK complex, which induces NF‐κB activation. Under the stimulation of Toll‐like receptor and interleukin‐1 (IL‐1), myeloid differentiation primary response 88 (MyD88)‐dependent signal transduction induces the recruitment of IRAK1/4 and TRAF6 to activate TAK1‐IKK complex, which then activates NF‐κB. In the noncanonical NF‐κB pathway, specific subsets of the TNFR superfamily are engaged with their ligands to induce the recruitment of TRAF2/3‐cIAP. After TRAF3 is ubiquitinated and degraded, NF‐κB‐induced kinase accumulates in the cytoplasm and then binds to IKKα, mediating p100 phosphorylation and ubiquitination‐dependent processing to produce p52/RelB heterodimers. In these processes, A20, cylindromatosis, and Otud7b play important roles in inhibiting ubiquitination of key molecules of NF‐κB pathways

In the canonical NF­κB pathway, the key event is the phosphorylation of IκB protein mediated by IKKs (Figure 2). The adaptor molecule, ubiquitin ligase, and protein kinase activate the IKK complex at different levels.^22^, ^26^ The canonical activation pathway of NF‐κB is stimulated by various factors, such as tumor necrosis factor receptor (TNFR) superfamily members, ligands of various cytokine receptors, pattern recognition receptors (PRRs, like Toll‐like receptor (TLR) ligands), as well as B‐cell receptor (BCR) and T‐cell receptor (TCR).^16^, ^27^, ^28^ After stimulation and upstream signal transduction, IKKs phosphorylate IκBs, which in turn are ubiquitinated by ubiquitin ligase and degraded by 26S proteasome, losing the ability to bind to NF‐κB. When IκB is degraded, NF‐κB is dissociated from the NF‐κB/IκBs complex and transferred into the nucleus, binds to the target gene promoter region on DNA, and initiate the transcription of the target gene.^5^, ^11^, ^29^ In this activation process, p50/RelA and p50/c‐Rel have mainly involved dimers. In addition, in the canonical NF‐κB pathway, one of the target genes activated is the gene encoding IκBα (*Nfkbia*), so IκBα is quickly re‐synthesized after degradation, and the newly synthesized IκBα can directly bind to NF‐κB in the nucleus. In this way, NF‐κB is dissociated from the κB‐binding site of DNA. The NF‐κB/IκBs complex is exported back to the cytoplasm to keep the original latent state, fulfilling the cycle of NF‐κB activation and inactivation. Therefore, the activation of the canonical NF‐κB pathway is usually robust and transient.^9^ The canonical NF‐κB pathway plays an important role in cell survival and proliferation, tumor cell epithelial to mesenchymal transformation (EMT), angiogenesis, cancer metastasis, and inflammation.

In the noncanonical NF‐κB pathway, the key event is the processing of the precursor protein p100 of NF‐κB2 rather than the degradation of IκBα (Figure 2). Compared with the canonical pathway with a wide variety of activators, the noncanonical pathway has relatively fewer activators. Noncanonical pathway selectively responds to specific stimuli, including ligands for part of TNF receptor family members, like lymphotoxin (LT) β receptor (LTβR), CD40, B‐cell activation factor family (BAFF), and receptor activator of NF‐κB (RANK).^30^, ^31^, ^32^, ^33^, ^34^ Under the action of stimuli, the corresponding receptor is activated, leading to degradation of TNF receptor‐associated factor 3 (TRAF3). The protein level of NF‐κB‐induced kinase (NIK) is mediated by TRAF3‐cellular inhibitor of apoptosis (cIAP) under the steady‐state condition. It is currently believed that NIK and IKKα mediate the conversion of p100 to p52, which are the key components of the noncanonical NF‐κB pathway.^35^ NF‐κB2 exists in the form of precursor p100 under the non‐stimulated condition. p100 functions as IκB protein, binding to RelB and retaining RelB in the cytoplasm.^36^ Together with IKKα, NIK mediates the phosphorylation of p100, which is further conjugated with ubiquitin chains mediated by β‐transducin repeats‐containing proteins (β‐TrCP) E3 ligase. Ubiquitinated p100 is processed to generate p52, forming p52/RelB heterodimer.^30^, ^32^, ^37^

Two NF‐κB pathways have shared characteristics: forming functional NF‐κB dimer and translocating the dimer into nuclear. The difference between these two pathways is that the activation of the canonical pathway is to rely on the degradation of IκBα; meanwhile, in the noncanonical NF‐κB pathway, processing of p100 into p52 does not only generate p52 but also liberate RelB from p100, forming p52/RelB dimer. The resynthesis of NIK is the key to start the downstream pathway, so the activating non‐standard NF‐κB pathway is much slower than the standard pathway.^21^ Optimizing target gene expression also requires the interaction of NF‐κB with other transcription factors, such as activator protein 1 (AP1), signal transducer and activator of transcription (STAT) family members, and interferon regulatory factors (IRFs).^38^, ^39^, ^40^

### 2.4 Regulation of NF‐κB pathway

As mentioned in the previous chapter 2.3, NF‐κB is often blocked in the cytoplasm by IκBs, keeping the entire pathway silent. However, once activated by stimuli (regardless of the various stimuli in the canonical pathway or a subset of specific stimuli in the noncanonical pathway), NF‐κB is activated to induce the production of pro‐inflammatory mediators and molecules leading to inflammation and activation and differentiation of the immune cell.^29^ If the NF‐κB activation is dysregulated, diseases such as chronic inflammation, tumors, and autoimmune diseases may occur. Therefore, NF‐κB is strictly regulated to maintain homeostasis.

First of all, as an inhibitory protein of NF‐κB, IκB plays a very important role in controlling the intensity and duration of NF‐κB activity. The target genes of NF‐κB include genes encoding IκB, such as *Nfkbia* (encoding IκBα) and *Nfkbie* (encoding IκBε). When the activated NF‐κB dimer enters the nucleus to bind to the κB‐binding site, target genes including *Nfkbia* are transcribed, and the newly generated IκBα and IκBε proteins bind and drive the NF‐κB dimer from the nucleus to the cytoplasm. This dynamic negative feedback mechanism is critical for keeping the activation of NF‐κB in check.^41^, ^42^, ^43^ Although both IκBα and IκBε have regulatory functions, they function in different ways. Compared with the rapid production and inhibition of IκBα, IκBε provides slower negative feedback regulation with delayed expression.^44^ In contrast, another member of the IκB family, IκBβ, directly binds to RelA and c‐Rel in the nucleus to counteract the inhibitory function of IκBα. When the hypo‐phosphorylated IκBβ binds to RelA and c‐Rel, the inhibitory effect mediated by IκBα is impaired, and the continuous transcription of the target gene is maintained.^24^, ^45^ In addition to driving NF‐κB back to the cytoplasm through the IκB protein, another way to downregulate NF‐κB activation is to degrade NF‐κB dimers in the nucleus directly. For example, elongin‐B‐elongin‐C‐cullin‐SOCS1 (ECS) is a ubiquitin ligase complex that promotes ubiquitination and subsequent degradation of RelA in the nucleus.^46^, ^47^, ^48^ Studies have also reported that the E3 ubiquitin ligase post‐synaptic density‐95, disks‐large, and zonula occludens‐1 and Lin‐11, Isl1 and Mec‐3 (LIM) domain protein 2 directly remove RelA from the DNA binding site and mediate its degradation to suppress NF‐κB transcription activity.^49^, ^50^ Protein inhibitor of activated STAT 1 (PIAS1) also moves to the promoter region of NF‐κB target genes after phosphorylation of IKKα, inhibiting the binding of RelA‐containing dimers to DNA.^51^, ^52^, ^53^

Secondly, the studies on NF‐κB signaling pathways reveal that ubiquitination is essential for NF‐κB activation. In the canonical NF‐κB pathway, NF‐κB activation mediated by multiple stimuli requires ubiquitination. For example, when interleukin‐1 receptor (IL‐1R) or TLR is activated, the ubiquitination of tumour necrosis factor receptor‐associated factor 6 (TRAF6) requires TRAF6‐regulated IKK activator 1 (TRIKA1, a dimeric ubiquitin‐conjugating enzyme complex, composed of ubiquitin‐conjugating enzyme 13 (Ubc13) and ubiquitin E2 variant 1Uev1A).^54^, ^55^ The function of TRIKA1 is to synthesize K63‐linked polyubiquitin chains on IKKγ and TRAF6.^54^, ^55^, ^56^ TRAF6 requires TRIKA2 (a complex composed of TAK1, TAB1, and TAB2) to activate IKK, and the activation of TRIKA2 requires ubiquitination of the adaptor protein TAB2/3.^55^, ^57^, ^58^ Similarly, when stimulation goes through TNFR, the activation of IKK depends on K63‐ubiquitination of receptor‐interacting protein 1 (RIP1), and this process is mediated by cIAP1/2.^59^ In the noncanonical NF‐κB pathway, p100 ubiquitination is the key event to regulate NF‐κB activation. β‐TrCP acts as a ubiquitin E3 ligase to mediate inducible p100 ubiquitination, another E3 ligase Fbxw7α requires glycogen synthase kinase 3 (GSK3)‐mediated phosphorylation to mediate ubiquitination and degradation of p100.^37^, ^60^ Another molecule of interest is deubiquitinase (DUB) A20. A20 contains the DUB domain and the C2‐C2 zinc finger E3 ubiquitin ligase domain, which inhibits K63 ubiquitination during the activation of NF‐κB, leading to the decomposition of the IKK complex. A20 also mediates the K48 ubiquitination of RIP1 after inhibiting K63 ubiquitination, causing the degradation of RIP1 and suppressing the signaling pathway induced by TNFα.^61^, ^62^, ^63^, ^64^ As a key enzyme that inhibits ubiquitination in the canonical NF‐κB pathway, A20 regulates NF‐κB activity by coordinating with another E3 lineage such as Itch.^65^ Meanwhile, the A20 encoding gene, *Tnfaip3*, is one of the NF‐κB target genes to mediate the inactivation of NF‐κB, suggestive of negative feedback regulation.^66^ Similar to A20, the tumor suppressor protein cylindromatosis (CYLD) is also a DUB involved in the regulation of NF‐κB activity. Its function is to remove K63 ubiquitin chain of key molecules upstream of IKK, including TRAF2/6 and IKKγ.^67^, ^68^, ^69^ In noncanonical NF‐κB pathway, ovarian tumor domain‐containing protein 7B (OTUD7B) functions as the DUB of TRAF3 to stabilize TRAF3 protein upon stimulation.^70^ OTUD7B deficiency leads to enhanced noncanonical NF‐κB activity. Additionally, OTUD 1 is also a DUB, which inhibits the ubiquitination of p100, leading to the stabilization of p100 under stimulation condition and steady state.^71^

Up to date, all the noncanonical NF‐κB‐activating stimuli activate NIK, which means that NIK is essential for noncanonical NF‐κB activation.^72^ Considering the importance of NIK, the regulatory factors of NIK are also considered to play an important role in the regulation of NF‐κB. As a negative regulator of NIK, TRAF3 binds to NIK through the N‐terminal region causing NIK degradation in a ubiquitination‐dependent way and keeping NIK protein at a very low level.^73^ This process also requires the participation of TRAF2 and ubiquitin E3 ligase cIAP1/2.^74^ This constitutive proteasome‐mediated degradation regulated by TRAF2, TRAF3, and cIAP1/2 inhibits NIK.^33^, ^74^ NIK is also regulated by TBK1. TBK1 mediates the phosphorylation of NIK at Ser862, which is located in the degradation domain of NIK, making the phosphorylated NIK unstable.^75^, ^76^ Moreover, TBK1 forms a ternary complex with TRAF family member‐associated NF‐κB activator (TANK), and TRAF2 and plays a role upstream of the NIK and IKK complex, a substitute for the receptor signaling complex for TRAF‐mediated NF‐κB activation.^77^ Kinase inactive TBK1 inhibits TANK‐mediated NF‐κB activation but does not block TNFα, IL‐1, or CD40‐mediated NF‐κB activation.^77^ IKKα also mediates the negative feedback regulation of NIK. IKKα phosphorylates the C‐terminus of NIK and causes NIK degradation to prevent excessive accumulation of NIK.^78^

In summary, most of the regulating mechanisms of the NF‐κB pathway are based on the regulation of key molecules or processes of this pathway, such as IKKs, NIK, ubiquitination. These mechanisms also indicate that the activation of NF‐κB is strictly regulated at multiple layers. Due to various stimuli and target genes involved in the NF‐κB pathway, the precise and comprehensive understanding of regulating mechanisms of the NF‐κB pathway requires additional intensive studies in vivo and in vitro.

## 3 NF‐ΚB AND INFLAMMATION

Inflammation is a protective response to infection and injury,^4^ which is usually beneficial and transient, but sometimes inflammation is also harmful. Excessive inflammation causes tissue damage and inflammation‐related diseases. As the central activator of a variety of pro‐inflammatory genes, NF‐κB plays an important role in innate and adaptive immune cells and inflammation.^5^, ^79^ Due to the diversity of inflammatory factors, including infectious and non‐infectious stimuli, the function of NF‐κB in inflammation is complicated.

### 3.1 NF‐κB regulates innate immune response and inflammation

The innate immune cells including macrophages, dendritic cells (DCs), and neutrophils, are required to produce inflammatory mediators and regulators to eliminate pathogens, while at the same time avoiding a sustained inflammatory response through negative feedback mechanisms.^80^ The PRRs expressed by these cells can detect various microbial components, so‐called pathogen‐associated molecular patterns (PAMPs). PRRs can also sense molecules released by necrotic cells and damaged tissues, called damage‐associate molecular patterns (DAMPs).^81^, ^82^ The PRRs on innate immune cells induce the expression of pro‐inflammatory cytokines such as TNF, IL‐1, IL‐6, IFN‐I, chemokines, and antimicrobial proteins and mediate an inflammatory response to eliminate pathogens and repair damaged tissue.^83^, ^84^ Different PRRs families have different structural characteristics and respond to different PAMPs and DAMPs. Five PRR families have been found in mammals, including TLRs, NOD‐like receptors (NLRs), RIG‐I‐like receptors (RLRs), C‐type lectin receptors (CLRs), and cytosolic DNA sensors.^83^, ^85^, ^86^, ^87^ The activation of the canonical NF‐κB pathway through these PRRs induces pro‐inflammatory cytokines, chemokines, and other inflammatory mediators.^82^, ^88^ Moreover, NF‐κB plays an important role in the signal transduction mediated by granulocyte‐macrophage colony‐stimulating factor (GM‐CSF) and myeloid progenitor cell differentiation.^89^ The development of innate immune cells from myeloid progenitor cells is specifically affected by different members of the NF‐κB transcription factor family.^90^

#### 3.1.1 NF‐κB functions in macrophage

Macrophages are the phagocytic innate immune cells against infection, and NF‐κB is crucial to regulate macrophage function. Macrophages can be activated by many PAMPs and DAMPs and secrete cytokines and chemokines.^4^ Activated macrophages can differentiate into different phenotypes, including M1 and M2 macrophages.^91^ M1 macrophages are characterized by producing pro‐inflammatory cytokines to promote inflammation, while M2 macrophages produce anti‐inflammatory cytokines to inhibit inflammation.^92^, ^93^ NF‐κB is a key transcription factor of M1 macrophages and is required for the expression of a large number of inflammatory genes.^91^ TLR signaling plays an important role in regulating the polarization of macrophages. A clear example is the TLR4 ligand lipopolysaccharide (LPS), which promotes the differentiation of macrophages to the M1 phenotype.^94^ Under LPS stimulation, TLR4 recruits toll‐interleukin 1 receptor domain‐containing adaptor protein (TIRAP) and Toll/IL‐IR domain‐containing adapter‐inducing IFN‐beta (TRIF)‐related adaptor molecule (TRAM) and further recruits MyD88 and TRIF for downstream signaling.^94^, ^95^ The signal from MyD88 activates the IRAK family (IRAK1 and IRAK4), which in turn stimulates TRAF6 and activate TAK1.^94^, ^96^, ^97^, ^98^ Subsequently, TAK1 mediates the phosphorylation and activation of IKKs, which in turn phosphorylates IκBα, leading to ubiquitin‐dependent IκBα degradation and NF‐κB activation, and the expression of pro‐inflammatory cytokines.^56^, ^99^, ^100^ TRIF‐dependent signaling pathways can mediate inflammatory cytokines by recruiting TRAF6 and RIP1 to activate the canonical NF‐κB pathway.^28^, ^54^, ^96^, ^101^ TRAF3 can also recruit TRIF‐dependent signaling pathways, activate TBK1 and IKKɛ and phosphorylate IRF3, and then induce the dimerization of IRF3, leading to the transcription of pro‐inflammatory cytokines and Type I IFNs.^28^, ^91^, ^102^ NF‐κB can also regulate other pathways to regulate the inflammatory function of macrophages. IKKβ can inhibit the activation of STAT1 in macrophages and suppress its inflammatory function.^103^ In general, NF‐κB is a key mediator of the inflammatory response of macrophages and mediates the pro‐inflammatory signaling functions. It was found in mice that if both c‐Rel and p50 proteins are defective, the innate immune response to bacterial sepsis will be impaired, and macrophages were able to exert normal immune functions.^104^

#### 3.1.2 NF‐κB regulates DC function

As primary antigen‐presenting cells (APCs), DCs present antigens to T cells and activate the adaptive immune response as a bridge to link innate immunity with adaptive immunity.^105^ Under the stimulation of PRRs, DCs sense infection and tissue damage through the canonical NF‐κB pathway and mature into APCs.^106^ DCs also express members of the TNFR superfamily such as CD40, LTβR, and RANK, which can stimulate the noncanonical NF‐κB pathway.^107^ It has been found that both canonical and noncanonical NF‐κB pathways regulated DC development and maturation.^108^ For example, the increase of RelB expression level is related to DC maturation, and RelB‐deficient DCs cannot induce antigen‐specific T‐cell responses in vitro and in vivo.^109^, ^110^ A recent study found that RelB‐deficient mice have spontaneous allergic airway inflammation, but the adoptive transfer of RelB‐sufficient DCs can reverse this phenotype.^111^ RelB is considered a component of the noncanonical NF‐κB transcription factor, but the canonical pathway NF‐κB is also critical to the RelB activity in DCs^112^, ^113^ since RelB activity in DCs is negatively regulated by IκBα and IκBε.^106^ In addition to RelB, targeted deletion of IKKβ in DCs can prevent the accumulation of non‐lymphoid tissue DCs in lymph nodes and impair the transformation of regulatory T cells.^114^ In short, it is clear that RelB plays an important role in the development and maturation of DCs, but it is necessary to explore the regulatory mechanism of other NF‐κB pathway components in DCs.

### 3.2 Functions of NF‐κB in adaptive immunity

Inflammation also involves adaptive immunity. After activation, T cells and B cells proliferate and differentiate into effector cells. These effector cells mediate different immune responses, including the secretion of cytokines, cytotoxic T lymphocyte response, and the production of antibodies by B cells.^29^ It is worthy of special attention that the activated CD4^+^ T cells can differentiate into effector T‐cell subsets with different functions, including Th1, Th2, Th9, Th17, Tfh, and regulatory T (Treg) cells.^115^, ^116^, ^117^ The differentiation of CD4^+^ T cells is regulated not only by the cytokines secreted by APCs and other innate immune cells but also by T‐cell intrinsic factors.^4^ Th1 cells produce pro‐inflammatory cytokines IL‐12 and Interferon‐γ (IFN‐γ) to activate macrophages and mediate the immune response to intracellular pathogens and participate in inflammation.^115^ Th2 cells release IL‐4, IL‐5, and IL‐13 and stimulate the response of mast cells, eosinophils and basophils to pathogens.^118^, ^119^ Th17 cells can produce IL‐17 and IL‐22 to recruit neutrophils and monocytes to the site of inflammation and mediate immune responses against pathogens or autoantigens.^117^ Treg cells are produced during the intrathymic development and can produce cytokines, such as IL‐10 and transforming growth factor‐β (TGF‐β), to suppress the immune response.^115^, ^117^, ^120^ Similar to CD4^+^ T cells, activated naive CD8^+^ T cells can also proliferate and differentiate into various effector and memory cell types, including T effector cells, T memory stem cells, and T central memory cells, which are responsible for the elimination of tumor cells and viral infections cells.^121^, ^122^, ^123^ While B lymphocytes develop in the bone marrow, they differentiate into plasma cells that can produce pathogen‐specific antibodies after activation.^124^ Memory B cell is formed in the germinal center after the initial infection. In the case of re‐infection, memory B cells quickly produce antibodies and play an important role in the secondary immune response.^125^

#### 3.2.1 NF‐κB in T cell

Many studies have proved that NF‐κB is necessary for the differentiation of effector T cells and the recall response of memory T cells.^126^, ^127^, ^128^, ^129^ The initial activation of primitive T cells through TCR and costimulatory signals depends on the canonical NF‐κB pathway.^130^ TCR activates the canonical NF‐κB pathway through the CARD‐containing MAGUK protein 1 (CARMA1)/Bcl10/membrane‐associated lymphoid tissue (MALT) 1 complex, which requires protein kinase C‐θ‐mediated phosphorylation of CARD‐containing MAGUK protein 1 (CARMA1).^131^, ^132^, ^133^ After T cell activation, some TNFRs are induced to mediate the activation of the noncanonical NF‐κB pathway.^134^ The noncanonical NF‐κB pathway plays a role in regulating the development of natural killer T cells and γδT cells, and this function is mediated by medullary thymic epithelial cells.^135^, ^136^, ^137^

CD4+ T cells differentiate into different effector cells after stimulation, thereby participating in various immune responses. In mice, inhibiting the activation of NF‐κB by expressing an anti‐degradation IκBα in T cells can reduce the differentiation of Th1 cells.^138^ The generation of Th1 cells also requires c‐Rel. The main function of c‐Rel is to mediate Th1 polarization cytokines in APC. Mice with c‐Rel deficiency have impaired Th1‐mediated immune response and impaired IFN‐γ production.^139^ The production of IFN‐γ relies on RelA; and another family member, RelB, also plays an important role in Th1 differentiation through regulating T‐bet.^140^ NF‐κB synergized with the IL‐4 to promote the development of Th2 cells by triggering the activation of the transcription factor STAT‐6.^141^, ^142^

An initial study found that T‐cell‐specific IKKβ‐deficient mice have impaired T‐cell activation and are completely resistant to experimental autoimmune encephalomyelitis, a Th17‐dependent autoimmune disease.^143^ More studies later proved that NF‐κB regulated Th17 cells. For example, the defect of RelA in DCs results in reduced production of IL‐1α, IL‐1β, and IL‐6 under LPS stimulation, which affects the Th17 differentiation.^144^, ^145^ In CD4^+^ T cells, c‐Rel mediates the expression of IL‐21, an important cytokine for Th17 and Tfh cell differentiation, upon TCR stimulation. Experiments have confirmed that c‐Rel‐deficient mice have defects in Th17 and Tfh differentiation.^146^ Another NF‐κB family member, p52, can promote the pathological function of Th17 cells in neuroinflammation by regulating GM‐CSF.^147^ In addition, as a key adaptor in TCR signaling, the lack of CARMA1 impairers Th17 development.^145^ The function of NF‐κB on Th9 cell differentiation is less known. A study found that OX40, a member of the TNFR family, activated TRAF6 and NIK in CD4^+^ T cells, which further activated the noncanonical NF‐κB pathway and promoted the production of Th9 cells.^148^

Tregs are crucial to prevent autoimmunity and chronic inflammation.^149^ Although NF‐κB is proved to promote T‐cell activation and effector T‐cell differentiation, NF‐κB is also involved in Treg generation. The Treg development in the thymus and the expression of the forkhead box P3 (Foxp3) depend on c‐Rel.^150^, ^151^, ^152^ In addition, RelA and c‐Rel, which are activated by canonical NF‐κB signals, participate in mTEC differentiation by regulating the transcription of RelB and also regulate the negative selection of autoreactive T cells and development of regulatory T cells.^153^ p105 deficiency can also make CD4+ T cells more resistant to Treg‐mediated suppression of inflammation.^154^ In the mice lacking canonical NF‐kB pathway components (including CARMA1, IKK, BCL10, and TAK1), it was found that fewer Tregs were produced.^155^ A significant decrease in Tregs' development was also observed in mice with a deletion in CYLD, the negative regulator of IKKs.^156^ It is worth noting that Ubc13 maintains the immunosuppressive function of Tregs through the canonical NF‐κB signaling pathway and prevents Tregs from acquiring an inflammatory phenotype.^157^ The development of Treg may also be affected by the ablation of non‐canonical NF‐kB signaling.^158^ For example, a study found a decrease of Treg in mice with NIK knockout.^159^

#### 3.2.2 NF‐κB and B cell

NF‐κB is essential for the development, survival, and function of B cells.^160^, ^161^ The activation of NF‐κB family members p52 and RelB and the upstream kinases NIK and IKKα can participate in the formation of germinal centers.^162^, ^163^, ^164^, ^165^, ^166^ The initiation and maintenance of germinal center (GC) require the cooperation of different cells, including B cells, Tfh cells, follicular regulatory T cells, macrophages and follicular DCs.^167^, ^168^ Under the stimulation of LTβR, the noncanonical NF‐κB pathway in stromal cells mediate the production of chemokines, including CXCL13 and CCL21, to provide migration signals to B cells and promote GC formation.^169^ This process also promotes the B cells proliferation with high‐affinity BCR and clearance of the low‐affinity B cells.^170^, ^171^ These high‐affinity B cells differentiate into plasma cells and memory B cells and initiate humoral immune responses.^172^ In RelB‐deficient mice, the induction of CXCL13 and CCL21 is reduced, leading to defects in the microstructure of the spleen and defects in the development and structure of lymphatic organs.^169^ In addition, recent studies have shown that in NIK‐knockout (KO) mice, the deficiency of NIK reduces the number of GC B cells and class‐switching.^173^, ^174^ It was found that deleting *nfkb2* and *Relb* genes in B cells of germinal centers would lead to the collapse of established germinal centers.^163^, ^169^, ^175^ In short, NF‐κB plays an important role in regulating B‐cell survival, differentiation, and maintenance of germinal centers.

### 3.3 NF‐κB is involved in inflammatory diseases

#### 3.3.1 Rheumatoid arthritis (RA)

RA is an autoimmune and inflammatory disease characterized by chronic inflammation caused by immune cells infiltrating into the synovium, which is associated with the destruction of cartilage and bone, and progressive joint destruction occurs over time.^176^, ^177^ Studies on animal models and human patients have confirmed that NF‐κB is an important inflammatory mediator in RA.^4^ Studies have found that NF‐κB is activated in the synovial tissue of a mouse arthritis model, and the activation of NF‐κB increases as the disease progresses.^178^ Similarly, in the early studies, NF‐κB activation was detected in the synovial tissue of RA patients, and it was determined that NF‐κB plays an important role in joint inflammation.^179^, ^180^, ^181^ The pro‐inflammatory effect of NF‐κB in RA has long been recognized; both the canonical and the noncanonical pathways are involved in different aspects of the pathogenesis of RA.^182^, ^183^ For example, inflammatory bone loss in RA patients involves the abnormal generation and activation of osteoclasts, and the occurrence of osteoclasts requires RANK, which can stimulate the canonical and noncanonical NF‐κB pathways in osteoclast precursor cells.^184^, ^185^, ^186^ In addition to the participation of osteoclasts, the pathogenesis of RA also involves many cell types, including innate immune cells, inflammatory T cells, B cells, and synovial fibroblasts.^187^ NF‐κB mediates the production of pro‐inflammatory cytokines (such as TNFα, IL‐1, and IL‐6) of innate immune cells, promotes the recruitment of inflammatory cells, and causes joint inflammation.^188^, ^189^ In addition, the excessive production of inflammatory cytokines and the induction of osteoclasts promote the destruction of bone and cartilage and aggravate disease progression.^190^, ^191^, ^192^ In particular, as a key component of noncanonical NF‐κB pathways, NIK is highly expressed in synovial endothelial cells of RA patients, and NIK enhances CXCL12 expression in endothelial cells to promote pathogenic angiogenesis and synovial inflammation.^193^, ^194^ Moreover, the study also found that NIK‐deficient mice have resistance to antigen‐induced arthritis caused by T cell.^195^, ^196^ As mentioned earlier, T cells are also involved in the pathogenesis of RA, especially Th17 cells, which can recruit synovial tissues, release pro‐inflammatory cytokines in synovial tissues, and induce synovitis.^130^, ^176^ The inflammatory factors required for Th17‐cell differentiation (such as IL‐1, IL‐6, and IL‐23) are induced by NF‐κB.^4^, ^176^, ^197^ B cells are also involved in the pathogenesis of RA. In addition to producing autoantibodies, B cells regulate other immune cells and produce cytokines. Studies have found that the level of the B‐cell‐activating factor cloning to the tumor necrosis factor family (BAFF) in serum and synovial fluid of RA patients is usually increased, which is associated with the severity of the disease, while BAFF mainly activates noncanonical NF‐κB pathway to facilitates survival and maturation of B cells.^198^, ^199^

Because NF‐κB functions many aspects of RA, inhibiting NF‐κB is a potential therapeutic target for RA.^199^, ^200^ It is well accepted that the biological agents such as anti‐TNFα‐neutralizing antibodies (etanercept, infliximab, and adalimumab, etc.) and anti‐IL‐6‐neutralizing antibody (tocilizumab) target NF‐κB pathway to prevent joint destruction of RA patients.^201^ Recent studies have found that iguratimod (IGU) is a potential treatment of RA. IGU is a new type of disease‐modifying antirheumatic drug, which inhibits the production of immunoglobulin by inhibiting NF‐κB without affecting the proliferation of B cells, the production of various inflammatory cytokines and osteoclasts.^202^, ^203^ In addition, salvianolic acid B and *Ephedra gerardiana* aqueous ethanolic extract have also been found to inhibit joint inflammation by suppressing NF‐κB in animal models.^204^, ^205^ Drugs targeting NF‐κB have been found to not only suppress inflammation but also effectively maintain bone mass and control the bone destruction of RA.^176^ Furthermore, the latest study tried to treat collagen‐induced arthritis mice with an injection of β‐arrestin‐2 (βArr2) adenovirus and found that βArr2 effectively reduced ankle joint inflammation by inhibiting the NF‐κB pathway and NLRP3 inflammasome.^206^ The latest clinical trials have also found that the activation of NF‐κB in RA patients treated with β‐d‐mannuronic acid is significantly reduced, as well as the levels of IL‐6 and TNFα in serum, reflecting the prospect of this treatment.^207^ The possibility of serious side effects caused by inhibiting NF‐κB should be considered since serious adverse reactions such as embryonic death have been reported.^208^, ^209^, ^210^, ^211^ We need to carry out further more basic and clinical research on the molecular mechanisms of RA and NF‐κB to find the best drugs for RA treatment.

#### 3.3.2 Inflammatory bowel disease (IBD)

IBD is a chronic, recurrent and non‐infectious gastrointestinal (GI) disease, including ulcerative colitis (UC) and Crohn's disease, accompanied by long‐term abdominal pain, diarrhea, and bloody stools.^212^, ^213^, ^214^ It is believed that the interaction between immunological, environmental, and genetic factors is regarded as the pathogenesis of IBD.^215^, ^216^, ^217^, ^218^, ^219^ In genetically susceptible individuals, when the balance of immune response to commensal bacteria is disturbed, the GI homeostasis is failed and the inflammation occurs.^214^ Both innate immunity and adaptive immunity play important roles in the pathogenesis of IBD; NF‐κB is involved in both aspects of immunity as an important inflammatory signaling pathway in the pathogenesis of IBD.^4^, ^215^ Early studies have reported that constitutive NF‐κB activation was found in the inflamed intestinal tissues of IBD patients.^220^, ^221^ In addition, *NFKB1* genes encoding p105 and p50 were found to be associated with IBD.^222^, ^223^, ^224^ Mice carrying knock‐in mutations of *NFKB1* to block the production of p105 can develop IBD‐like intestinal inflammation.^154^ Similarly, *Nfkb2* also promotes the expression of RelA‐driven pro‐inflammatory genes in the intestinal epithelium and aggravates inflammatory cell infiltration and colon tissue damage.^225^ Consistent with these findings, decoy oligodeoxynucleotides targeting the DNA‐binding activity of NF‐κB showed potential therapeutic prospects in mice model.^226^ Genetic defects of negative regulators of NF‐κB pathway can promote colon inflammation and even cancer. For example, in a colitis‐related cancer model, compared with the control group, CYLD‐deficient mice are more susceptible to colonic inflammation, and the incidence of tumors is significantly increased.^227^ Deletion of A20 in intestinal epithelial cells and myeloid cells induces ileitis and severe colitis.^228^ Although NF‐κB participates in the pathogenesis of IBD, its functions in innate immune cells and epithelial cells are different. Loss of IKKβ in myeloid cells inhibits pro‐inflammatory cytokines expression, attenuating colitis and colitis‐related cancers.^229^ Contrary to the pro‐inflammatory effect in myeloid cells, NF‐κB has a protective effect in intestinal epithelial cells and is a key regulator of epithelial integrity and intestinal immune homeostasis.^230^ Using the conditional KO of IKKβ and IKKγ in intestinal epithelial cells, it shows the excessive and abnormal immune response in the intestine.^229^, ^230^ It has been confirmed that the IKKβ‐dependent gene expression in the intestinal epithelium is essential for intestinal immune homeostasis by promoting mucosal immunity and limiting chronic inflammation.^231^ In the intestinal epithelium, NF‐κB not only plays a protective role but also participates in the regulation of the intestinal tight junction barrier. The latest research shows that the destruction of matrix metalloproteinase‐9 (MMP‐9)‐induced Caco‐2 intestinal epithelial tight junction barrier is regulated by the NF‐κB pathway.^232^ This finding suggests that the function of NF‐κB in the intestinal epithelium is complex and to be further studied. As a mediator between the immune response and the intestinal commensal microbiota, TLRs also play a key role in maintaining intestinal homeostasis.^233^ The enhanced expression of TLRs leads to a hyper‐activated downstream signal cascade including NF‐κB and increased inflammatory cytokines and IBD.^214^, ^233^ Under normal physiological conditions, the expression level of TLR4 in the intestinal epithelium is low to maintain the integrity of the mucosa and protect against invading bacteria; however, its expression is upregulated in IBD, activating the downstream cascade and leading to inflammation.^233^, ^234^

The current treatment strategy for IBD is to control mucosal inflammation and inhibit overwhelming immune responses.^235^, ^236^ Commonly used drugs include 5‐aminosalicylic acid, glucocorticoid, immune‐suppressor, and biological agents.^237^ A biological agent that has attracted much attention is the anti‐TNF antibody infliximab, which has shown good therapeutic effects in IBD.^238^, ^239^ The success of infliximab indicates that inhibiting the inflammatory target genes of NF‐κB is one of the effective treatment strategies for IBD. In addition, for glucocorticosteroid treatment, the activity of NF‐κB is decreased in UC patients who respond to the treatment, which may be due to the increase in the level of glucocorticoid receptors that regulate the activity of NF‐κB.^240^ There are also some medicines in the development stages that show certain therapeutic prospects. A recent study found that butyrolactone‐I reduces the production of IL‐1, IL‐6, and TNFα by inhibiting the TLR4/NF‐κB and mitogen‐activated protein kinases (MAPK) signaling pathways and reduces the inflammatory response of dextran sulfate sodium‐induced colitis in mice.^241^ Another study found that a type of 2,3‐dihydro‐flavonoid has a beneficial effect on 2,4,6‐trinitrobenzene sulfonic acid‐induced colitis, possibly by inhibiting NF‐κB p65 phosphorylation to reduce inflammatory mediators produce.^242^

#### 3.3.3 Atherosclerosis

Atherosclerosis is a multi‐step inflammatory process in the arterial wall, characterized by the accumulation of low‐density lipoprotein (LDL) particles and immune cells in the subendothelial space. Both innate immunity and adaptive immunity are involved in this disease.^243^, ^244^ NF‐κB regulates the expression of genes involved in the pathogenesis of atherosclerosis.^245^, ^246^ The first step in atherosclerosis is the injury and activation of endothelial cells. In endothelial cells, NF‐κB mediates the expression of inflammatory cytokines, chemokines, and cell adhesion molecules and promotes the recruitment of monocytes to the arterial intima.^4^, ^247^, ^248^ Then, the recruited monocytes differentiate into macrophages, and after ingesting LDL particles, they will eventually become lipid‐rich foam cells, which is a hallmark of the arterial lesion.^245^ In foam cells, NF‐κB is activated, pro‐inflammatory cytokines are secreted to enhance inflammation; the vascular smooth muscle cells are recruited to the inflamed sites, and finally atherosclerosis is formed.^19^, ^245^ Considering the pro‐inflammatory function of NF‐κB and inflammation play crucial roles in the process of atherosclerosis, inhibiting NF‐κB may be a target for the prevention and treatment of atherosclerosis. Inhibition of NF‐κB reduces the expression of inflammatory cytokines and chemokines and suppresses the induction of adhesion molecules in endothelial cells, to reduce the recruitment of macrophages to plaques and vascular inflammation. In an apolipoprotein E (ApoE)‐deficient mouse model fed by a high‐cholesterol diet, NF‐κB was inhibited by ablation of IKKγ or increased expression of IκBα, and atherosclerotic plaque formation was significantly reduced.^249^ Studies have also found that in macrophages, the overexpression of trans‐dominant and non‐degradable forms of IκBα specifically inhibits NF‐κB activation, which can reduce lipid load and foam cell formation.^250^ Similarly, one study has shown that the lack of IκBα in myeloid cells may promote atherosclerosis by reducing leukocyte recruitment to plaque.^251^ However, the role of macrophage‐specific NF‐κB in the development of atherosclerosis is still incompletely known.^245^

Targeting NF‐κB‐dependent inflammation is a very promising strategy for atherosclerosis treatment. A large clinical trial used anti‐IL‐1β antibody (canakinumab) for anti‐inflammatory treatment in patients with atherosclerosis and previous myocardial infarction. The results showed that, compared with placebo, the recurrence rate of cardiovascular events was significantly reduced in the group with treatment.^252^ The results of this clinical trial demonstrate the effectiveness of anti‐inflammatory treatments in the management of atherosclerotic diseases. Recent studies have also confirmed the therapeutic value of NF‐κB as a target for atherosclerosis using animal models. The traditional Chinese medicine *Qing‐Xue‐Xiao‐Zhi* formula inhibits TLR4‐mediated NF‐κB pathway, affects cholesterol metabolism and promotes lipid efflux, and inhibits macrophage‐mediated inflammation, which has a therapeutic effect on atherosclerosis.^253^ Another clinical trial has also found that crocin may reduce the expression of lectin‐like oxidized LDL receptor 1 and NF‐κB by increasing expression of sirtuin 1 and 5′‐adenosine monophosphate‐activated protein kinase (AMPK), demonstrating the potential therapeutic value of coronary heart disease.^254^ The latest research has found that *Paeonia lactiflora* extract also exhibits a certain therapeutic potential for atherosclerosis. In vitro *P. lactiflora* extract can inhibit TNFα‐induced nuclear translocation of NF‐κB p65 from the cytoplasm and NF‐κB activity; in vivo oral administration of *P. lactiflora* extract can improve TNFα‐induced macrophages activation. The infiltration of immune cells to the vascular endothelium and the expression of *Il6* and *Tnfa* in the mouse aorta contribute to the early stage of atherosclerosis.^255^

#### 3.3.4 Corona virus disease 2019 (COVID‐19)

In the past 2 years, COVID‐19 has had a catastrophic impact on the health and lives worldwide. According to the current vaccination situation and drug development, this impact will continue for a certain time. COVID‐19 patients show a wide range of clinical features, including cough and fever, and even some patients develop septic shock, acute respiratory distress syndromes, and multiple organ failure.^256^, ^257^, ^258^ Compared with asymptomatic patients or mildly symptomatic patients, the clinical manifestations of severe cases suggest excessive activation and imbalance of systemic inflammation.^256^, ^259^ In particular, cytokine storm syndrome (CSS) occurs in critically ill patients, with significantly increased cytokine and chemokine.^260^, ^261^, ^262^ The overwhelming production of these cytokines is related to the severity of COVID‐19, and the NF‐κB signaling pathway plays an important role in it.^263^, ^264^ Activated NF‐κB upregulates the production of inflammatory cytokines (such as TNFα, IL‐1β, IL‐6, and IL‐8), which is essential for a comprehensive cytokine storm.^265^, ^266^ Multiplication of severe acute respiratory syndrome coronavirus 2 (SARS‐CoV‐2) promotes the production and accumulation of dsRNA.^267^ Protein kinase R (PKR) can trigger the innate immune response and terminate the translation process to prevent replication of the virus in infected cells. PKR combined with dsRNA can activate IKK, trigger the degradation of IκBα and IKKβ, and activate canonical NF‐κB pathway.^268^, ^269^ SARS‐CoV‐2 spikes and nucleocapsid (N) protein induced NF‐κB activation, which in turn significantly increased the expression of pro‐inflammatory cytokines. The spike protein subunit 1 of SARS‐CoV‐2 has been identified as an effective cytokine storm inducer in COVID‐19.^270^ This subunit has a high binding affinity to the angiotensin‐converting enzyme 2 receptor and can activate NF‐κB to produce cytokines.^271^ In addition, SARS‐CoV‐2 N protein can also promote NF‐κB activation and increase the expression of cytokines by recruiting TAK1 and IKK complexes after binding to viral RNA.^262^ SARS‐CoV‐2 mediates inflammation by activating NF‐κB, so the development of drugs that inhibit NF‐κB is currently considered to be one of the potential treatment strategies for COVID‐19.^272^ A clinical trial used the BTK inhibitor acalabrutinib to treat severe COVID‐19 patients, and the result showed that C‐reactive protein and IL‐6 levels were reduced, and patients' oxygen saturation improved.^273^ Another study used diosmectite in the SARS‐CoV‐2 model, as diosmectite can bind to SARS‐CoV‐2 components and inhibit NF‐κB activation and CXCL10 secretion and inhibit downstream inflammation, indicating the potential value of diosmectite for COVID‐19‐related diarrhea.^274^ It is worth noting that traditional Chinese medicine has shown considerable prospects in the treatment of COVID‐19. A clinical study found that Yindan Jiedu granules may inhibit the production of inflammatory cytokines by targeting the NF‐κB pathway, thereby shortening the course of COVID‐19 and delaying its progression.^275^ In in vitro experiments, it was also found that another traditional Chinese medicine, Liu Shen capsule, can inhibit SARS‐CoV‐2 infection by downregulating cytokine‐induced virus expression and regulating the activity of the NF‐κB/MAPK signaling pathway.^276^ Despite the intensive efforts and large‐scale drug screening, an effective and safe antiviral treatment plan has not been developed. Nucleotide analogs inhibit RNA‐dependent RNA polymerase (RdRp) to suppress dsRNA‐induced NF‐κB activation and prevent virus replication. One example is remdesivir, which shows a certain clinical effect, compared to a placebo.^277^ In addition, the latest drug development is the oral antiviral drugs molnupiravir and paxlovid, which have shown good treatment efficiency in clinical tries.^278^, ^279^ As an important inflammation‐related pathway, NF‐κB is still a potential therapeutic target for COVID‐19 treatment, especially for critically ill patients and CSS.

## 4 NF‐ΚB AND CANCER

### 4.1 Activation of NF‐κB in cancer

*v‐Rel* is an oncogene of avian Rev‐T retrovirus, isolated from a turkey liver lymphoma by Theilen and Robinson in 1958,^280^ which is the earliest evidence that NF‐κB is related to cancer. However, the carcinogenicity of the *rel* gene is only found in birds, not in humans. Although the gene *rel* is not a bona fide oncogene, the constitutive‐activated NF‐κB has been examined in most tumors, which participates in a variety of cancer‐related biological procedures.^16^

In most cancers, the activation of NF‐κB is enhanced, and this enhancement in NF‐κB activation is often due to increased stimuli of the NF‐κB pathway, such as increased TNFα and IL‐1 in the tumor microenvironment.^281^, ^282^ On the other hand, NF‐κB has a certain tumor‐suppressing function confirmed using tumor cell line and mouse models but not fully demonstrated in human cancers yet.^283^, ^284^ Mutations of NF‐κB are found in many cancers, although the mutation frequency of RelA and RelB is much lower than that of REL, p50, and p52.^7^ It has been reported that REL gene amplification in lymphoma leads to an increased expression of REL protein, as well as a C‐terminal truncated p100 protein that lacks the ANK repeat inhibitory sequence.^285^, ^286^, ^287^ In addition to NF‐κB family proteins, mutations have been found in the core components and regulators of the NF‐κB pathway, which affect both canonical and noncanonical NF‐κB activation. For example, studies have identified a loss‐of‐function mutation of IκB family proteins in lymphoma, glioblastoma, and nasopharyngeal carcinoma.^288^, ^289^, ^290^, ^291^ Meanwhile, studies have also reported that mutations of IKKα and IKKβ have been found in several cancers,^292^, ^293^, ^294^ although the mutation of IKK is relatively rare. The reason may be that IKK also participates in many other signaling pathways except for NF‐κB.^295^ The functional versatility of IKK may reduce the possibility of mutations. Mutations in critical regulating molecules that affect the activation and function of NF‐κB are identified in tumor cells. Considering NF‐κB as a family of the conserved transcription factor, the most common mutations that enhance NF‐κB signaling may occur in the upstream molecules and downstream target genes.^7^ In diffuse large B‐cell lymphoma (DLBCL) and multiple myeloma (MM), proteins such as CD79 and MyD88 have been found to activate NF‐κB through gain‐of‐function mutations. It was also found that loss‐of‐function mutations of CYLD, A20, TRAF3, and other negative regulators also cause abnormal activation of NF‐κB.^296^, ^297^, ^298^, ^299^, ^300^, ^301^ Similarly, TNFα coded by an NF‐κB target gene *TNFA* acts as a strong activator of NF‐κB, functions in many tumor cells with constitutive activation of NF‐κB in an autocrine manner (usually stromal).^301^, ^302^ Taking specific tumor types as an example, DLBCL is the most studied malignant tumor with NF‐κB mutation.^297^ Studies have shown that mutations in the three proteins (REL, IκB, p300) are involved in the NF‐κB activation in human B‐cell lymphoma cell line RC‐K8.^287^, ^288^ Most B‐cell malignancies not only have an oncogenic driving force activated by mutated NF‐κB but also have mutations in many other pathways. The subtype of DLBCL lymphoma with the activation of the canonical NF‐κB pathway is named by the activated B‐cell (ABC) subtype. Meanwhile, the abnormal activation of the noncanonical NF‐κB pathway is also detected more in other DLBCL subtypes.^299^, ^300^ The mutations in the NF‐κB signaling pathway involved in cancers are summarized in Table 1.

**TABLE 1:** Mutations in the nuclear factor of κ‐light chain of enhancer‐activated B cells (NF‐κB) pathway identified in cancers

**Cancer type**	**Protein**	**Gene**	**Mutation type**	Effect	Ref.
**Solid**					
Bladder cancer	TRAF2/3	*TRAF2/3*	Mutations, deletions, amplifications	Decrease	^7^, ^426^
	TRAF4	*TRAF4*	Mutations, amplifications	Increase	^7^, ^426^
Breast cancer	TRAF4/5/6	*TRAF4/5/6*	Mutations, amplifications	Increase	^7^, ^426^
Cervical cancer	p50/p105	*NFKB1*	Point mutations	Increase	^427^
	TRAF3	*TRAF3*	Mutations, deletions, amplifications	Decrease	^7^, ^426^
Colon cancer	TRAF1	*TRAF1*	Point mutations	Increase	^428^
	TRAF6	*TRAF6*	Mutations, amplifications	Increase	^7^, ^426^
Cylindromatosis	CYLD	*CYLD*	Mutations, deletions	Decrease	^69^
Esophageal cancer	TRAF4/5/6	*TRAF4/5/6*	Mutations, amplifications	Increase	^7^, ^426^
Gastric cancer	p50/p105	*NFKB1*	Point mutations	Increase	^3^
	MYD88	*MYD88*	Mutations, deletions	Increase	^299^, ^429^
	TRAF1	*TRAF1*	Point mutations	Increase	^7^, ^426^
	TRAF2/3	*TRAF2/3*	Mutations, deletions, amplifications	Decrease	^7^, ^426^
	TRAF6	*TRAF6*	Mutations, amplifications	Increase	^7^, ^426^, ^430^
Glioblastoma	IκBα	*NFKBIA*	Mutations, deletions	Decrease	^290^, ^431^
Head and neck cancer	TRAF2/3	*TRAF2/3*	Mutations, deletions, amplifications	Decrease	^7^, ^426^
	TRAF6	*TRAF6*	Mutations, amplifications	Increase	^7^, ^426^
Liver cancer	p50/p105	*NFKB1*	Point mutations	Increase	^3^
	TRAF5/6	*TRAF5/6*	Mutations, amplifications	Increase	^7^, ^426^
Lung cancer	TRAF3	*TRAF3*	Mutations, deletions, amplifications	Decrease	^7^, ^426^
	TRAF4/5/6	*TRAF4/5/6*	Mutations, amplifications	Increase	^7^, ^426^
Melanoma	TRAF1	*TRAF1*	Point mutations	Increase	^426^, ^432^
	TRAF2/3	*TRAF2/3*	Mutations, deletions, amplifications	Decrease	^7^, ^426^
	TRAF4/5	*TRAF4/5*	Mutations, amplifications	Increase	^7^, ^426^
Nasopharyngeal carcinoma	IκBα	*NFKBIA*	Mutations, deletions	Decrease	^291^
	A20	*TNFAIP3*	Point mutations	Decrease	^291^
	CYLD	*CYLD*	Mutations, deletions	Decrease	^69^
	TRAF3	*TRAF3*	Mutations, deletions, amplifications	Decrease	^7^, ^426^
Ovarian cancer	p50/p105	*NFKB1*	Point mutations	Increase	^3^
	TRAF2/3	*TRAF2/3*	Mutations, deletions, amplifications	Decrease	^7^, ^426^
	TRAF4/5/6	*TRAF4/5/6*	Mutations, amplifications	Increase	^7^, ^426^
Pancreatic cancer	TRAF4	*TRAF4*	Mutations, amplifications	Increase	^7^, ^426^
Prostate cancer	IKKβ	*IKBKB*	Point mutations	Increase	^293^
	TRAF2	*TRAF2*	Mutations	Decrease	^7^, ^426^
	TRAF5/6	*TRAF5/6*	Mutations, amplifications	Increase	^7^, ^426^
Uterine cancer	TRAF2/3	*TRAF2/3*	Mutations, deletions, amplifications	Decrease	^7^, ^426^
	TRAF4/5/6	*TRAF4/5/6*	Mutations, amplifications	Increase	^7^, ^426^
**Hematologic malignancy**					
Chronic lymphocytic leukemia	IκBε	*NFKBIE*	Deletions, point mutations	Decrease	^433^
	BCL3	*BCL3*	Translocations	Increase	^434^
Chronic myelogenous leukemia	TRAF1	*TRAF1*	Point mutations	Increase	^435^
Diffuse large B‐cell lymphoma	p52/p100	*NFKB2*	C‐terminal truncations	Increase	^285^
	REL	*REL*	Point mutations, amplifications,	Increase	^287^
	REL	*REL*	Truncations	Decrease	^287^
	IκBα	*NFKBIA*	Mutations, deletions	Decrease	^285^
	IKKβ	*IKBKB*	Point mutations	Increase	^293^
	CD79A/B	*CD79A/B*	Point mutations	Increase	^436^
	BCL10	*BCL10*	Point mutations, chromosomal translocations	Increase	^437^
	LUBAC	*HOIP, HOIL, SHARPIN*	Point mutations	Increase	^438^
	MALT1	*MALT1*	Chromosomal translocations, point mutations, amplifications	Increase	^439^
	A20	*TNFAIP3*	Point mutations	Decrease	^440^
	CARD11	*CARMA1*	Chromosomal translocation; point mutation	Increase	^441^
	p300	*EP300*	Deletions	Decrease	^442^, ^443^
	TRAF2	*TRAF2*	Mutations	Decrease	^444^
	TRAF3	*TRAF3*	Mutations, deletions, amplifications	Decrease	^7^, ^426^
	CBP	*CREBBP*	Deletions	Decrease	^442^, ^443^
	MYD88	*MYD88*	Mutations, deletions	Increase	^299^
Hodgkin lymphoma	NIK	*MAP3K14*	Gene fusion, point mutations	Increase	^31^
Leukemia	CARD11	*CARMA1*	Chromosomal translocation; point mutation	Increase	^441^
	p300	*EP300*	Deletions	Decrease	^442^, ^443^
	CBP	*CREBBP*	Deletions	Decrease	^442^, ^443^
Mantle cell lymphoma	TRAF2	*TRAF2*	Mutations	Decrease	^444^
Marginal zone lymphoma	TRAF3	*TRAF3*	Mutations, deletions, amplifications	Decrease	^7^, ^426^
Multiple myeloma	RELA (p65)	*RELA*	Point mutations	Increase	^445^
	IκBβ	*NFIKBB*	Point mutations	Decrease	^446^
	IKKβ	*IKBKB*	Point mutations	Increase	^293^
	NIK	*MAP3K14*	Gene fusion, point mutations	Increase	^446^
	CYLD	*CYLD*	Mutations, deletions	Decrease	^69^
	TRAF3	*TRAF3*	Mutations, deletions, amplifications	Decrease	^7^, ^426^
B‐cell lymphoma	CD79A/B	*CD79A/B*	Point mutations	Increase	^436^
	p52/p100	*NFKB2*	C‐terminal truncations	Increase	^285^
	BCL10	*BCL10*	Point mutations, chromosomal translocations	Increase	^437^
	MALT1	*MALT1*	Chromosomal translocations, point mutations, amplifications	Increase	^439^
	A20	*TNFAIP3*	Point mutations	Decrease	^440^
	CARD11	*CARMA1*	Chromosomal translocation; point mutation	Increase	^441^
T‐cell lymphoma	p52/p100	*NFKB2*	C‐terminal truncations	Increase	^285^
Waldenström's macroglobulinemia	MYD88	*MYD88*	Mutations, deletions	Increase	^447^
	TRAF3	*TRAF3*	Mutations, deletions, amplifications	Decrease	^7^, ^426^

Abbreviations: Bcl3, B‐cell leukemia/lymphoma 3; CARD11, caspase recruitment domain‐containing protein 11; CARMA1, CARD‐containing MAGUK protein 1.; CBP, CREB binding protein; CYLD, cylindromatosis; HOIL, heme‐oxidized IRP2 ubiquitin ligase; HOIP, HOIL‐1L‐interacting protein; IKKβ, IκB kinase β; IκB, inhibitor of NF‐κB; LUBAC, linea ubiquitin assembly complex; MALT1, mucosa‐associated lymphoid tissue lymphoma translocation protein 1; MyD88, myeloid differentiation primary response gene 88; NIK, NF‐κB‐induced kinase; SHARPIN, shank‐associated RH domain interactor; TRAF, TNF‐R‐associated factor.

### 4.2 NF‐κB and cancer cell survival and proliferation

One important function of the NF‐κB is to regulate cell survival. In tumor cells, the activation of NF‐κB usually leads to impaired cell apoptosis.^303^ The deletion or inactivation of genes encoding key molecules in the NF‐κB pathway promotes cell apoptosis. For example, inactivation of the *v‐Rel* oncogene leads to apoptosis of transformed lymphocytes, and KO of the *Rela* gene leads to apoptosis of mouse fibroblasts and embryonic hepatocytes under the TNFα stimulation.^210^, ^304^ NF‐κB target genes encode anti‐apoptotic molecules such as Bcl2, Bcl‐xL, and inhibitor of apoptosis (IAP). Upregulation of NF‐κB activity has been detected in various tumor cells, and the expression of target genes of anti‐apoptotic NF‐κB is also increased.^7^ It is noteworthy that NF‐κB is not only anti‐apoptotic, the activation of NF‐κB is also required for cell apoptosis. Studies have found that inhibition of NF‐κB reduced the apoptosis induced by drugs in many tumor cells.^305^, ^306^, ^307^ Therefore, NF‐κB may also be a cell type‐specific repressor of anti‐apoptotic genes or an activator of pro‐apoptotic genes.^308^, ^309^, ^310^ In addition, the recent researches suggest that many molecules (such as various anti‐cancer drugs, non‐coding RNA, microRNA, etc.) regulate the apoptosis of cancer cell through the NF‐κB pathway and other coordinated pathways (such as AMPK, AKT, glycogen synthase kinase 3, metabolism pathways).^311^, ^312^, ^313^, ^314^, ^315^, ^316^, ^317^

In addition to apoptosis, proliferation is also the fundamental cellular procedure. NF‐κB activates genes that regulate cell proliferation, such as cyclin D1/D2/D3.^318^, ^319^, ^320^ In addition to cyclins, NF‐κB also regulates cell proliferation by induction of critical enzymes. The expression of E3 ubiquitin ligase mouse double minute 2 protein is NF‐κB‐dependent, which affects the stability of p53 and cell proliferation.^321^ In inflammatory immune cells such as macrophages and neutrophils, the activation of NF‐κB activates the expression of inflammatory cytokines such as TNFα, IL‐1β, and IL‐6, thereby promoting the proliferation of malignant cells and tumor stromal cells.^16^, ^322^, ^323^ Taken together, it has been well‐accepted that NF‐κB plays an important role in the regulation of tumor cell proliferation and apoptosis, and its functions and mechanisms are multi‐faceted.

### 4.3 NF‐κB and tumorigenesis

The journal from tumorigenesis to tumor progression is a long‐term procedure controlled by multiple factors. Most tumors usually require multiple gene mutations.^324^ Inflammation and NF‐κB affect the tumor occurrence by promoting the production of reactive oxygen species and reactive nitrogen species, leading to DNA damage and carcinogenic mutations.^282^ Chronic inflammation and NF‐κB also cause chromosomal instability, aneuploidy, and epigenetic changes, resulting in the occurrence and progression of tumors.^282^ In addition, one of the mechanisms by which NF‐κB promotes tumorigenesis is that when cells with damaged DNA strands enter the cell cycle, NF‐κB activation integrates mutations into the two DNA strands and transmits them to daughter cells.^325^, ^326^ Another mechanism by which NF‐κB stimulates tumorigenesis is to induce mutant‐related enzymes such as activation‐induced cytidine deaminase that deaminates cytosine residues and causes the conversion of cytosine to thymine, increasing mutations probability.^327^ Furthermore, by preventing p53‐dependent apoptosis, NF‐κB activation increases the number of DNA‐damaged cells that accumulate oncogenic mutations.^328^ In addition to gene mutation, viral infection is the risk factor leading to tumorigenesis, such as hepatitis B virus (HBV), human papillomavirus, and Epstein–Barr virus (EBV), and so forth. Some viruses promote tumorigenesis through chronic NF‐κB‐dependent inflammation or the continuous activated transcriptional activity induced by viral genes. Viruses also directly encode NF‐κB‐stimulating factors. EBV that causes B‐cell lymphoma and nasopharyngeal carcinoma encode LMP1 protein that functions as CD40 homolog to prevent apoptosis of EBV‐infected B‐cell by upregulating NF‐κB activation.^329^ Kaposi's sarcoma‐associated herpesvirus that causes sarcoma and lymphoma encodes viral FLICE inhibitory protein (vFLIP) that hijacks both canonical and noncanonical NF‐κB pathways promoting cell survival and proliferation.^329^ Besides direct transcribing cell proliferation‐related genes, NF‐κB also induces the expression of proinflammatory cytokines such as IL‐1β, IL‐6, and TNFα, which play a key role in the NF‐κB‐dependent tumor cell proliferation.^330^ Meanwhile, NF‐κB also regulates apoptosis of tumor cells. NF‐κB blocks the death of tumor cells induced by the activation of oncogenes Ras.^331^ NF‐κB‐dependent Rac guanosine triphosphatase effectively inhibits the p53‐independent apoptosis response induced by high levels of Ras activity. Rac mutants that could not activate NF‐kB are defective in inhibiting Ras‐induced apoptosis. In particular, under hypoxic conditions, NF‐κB enhances the expression of hypoxia‐inducible factor 1α, thereby enhancing the early survival of tumor cells.^332^, ^333^

**FIGURE 3:**
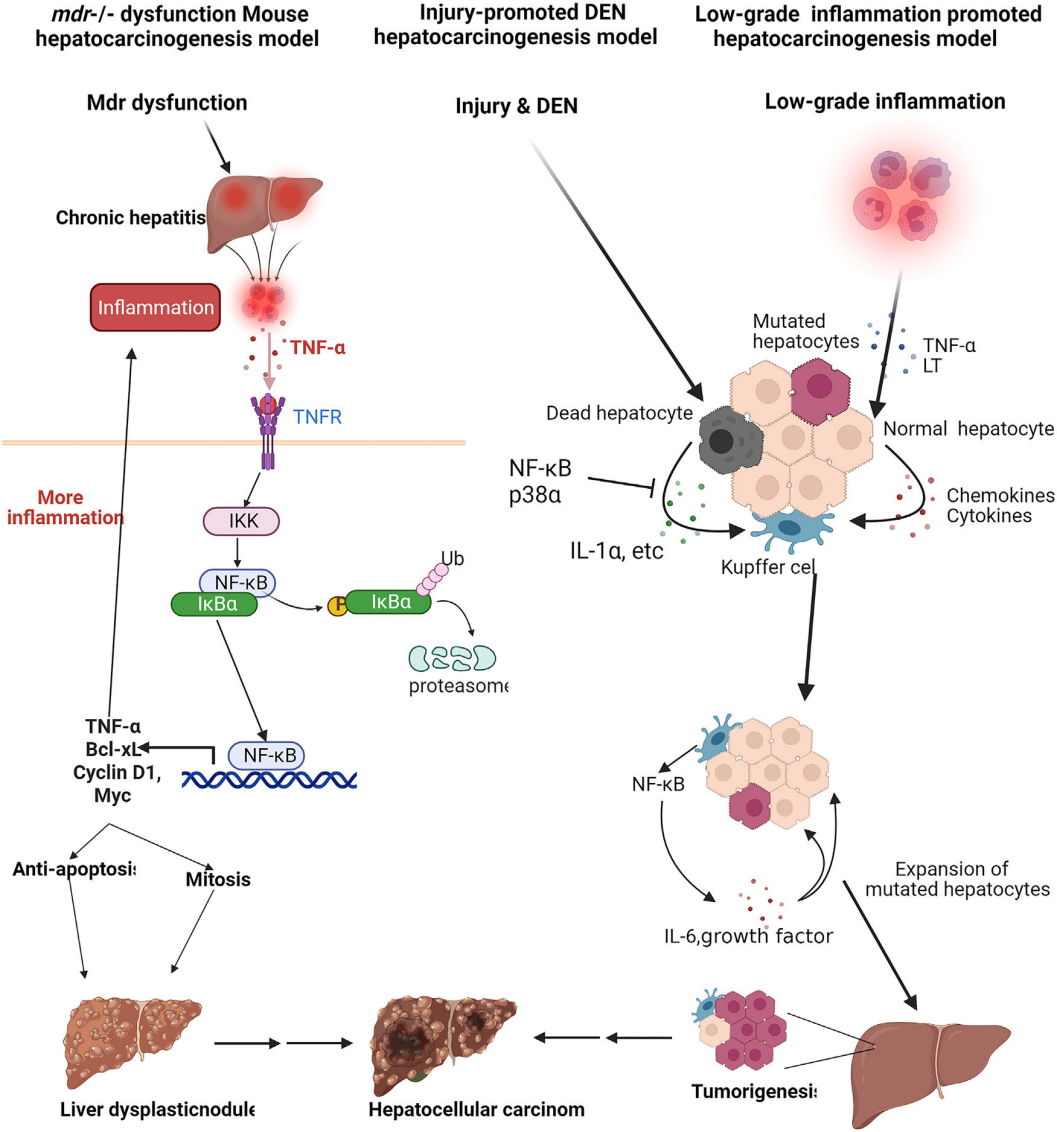
NF‐κB signaling pathway in tumorigenesis. Schematic diagram of the mechanism of gene mutation, exogenous carcinogenic stimulation, and inflammation leading to liver cancer. In the mice model, the deletion of *Mdr* gene leads to the accumulation of bile acids and induces chronic inflammation of the liver. After inflammatory cells migrate to the liver matrix, they release TNFα and invade the liver parenchymal cells between the liver epithelium. TNFα activates the NF‐κB signaling pathway, resulting in the expression of apoptosis‐inhibiting proteins, pro‐proliferation proteins and TNFα and promotes the development of dysplastic nodules into hepatocellular carcinoma. In the damage‐promoted diethylnitrosamine liver cancer model, Kupffer cells are activated by IL‐1α released by dead liver cells. In the low‐grade inflammation‐promoting hepatocellular carcinoma model, the hepatocytes with activated NF‐κB produce cytokines and chemokines. These cytokines and chemokines activate Kupffer cells. In these two models, activated Kupffer cells produce cytokines and growth factors, which promote the expansion of mutated liver cells and the development of liver cancer

NF‐κB is important for tumor angiogenesis.^334^ Among the singling pathways contributing to tumor angiogenesis, NF‐κB is critical to activate angiogenesis‐related genes. Study has confirmed that B7‐H3 activates NF‐κB pathway to upregulate the expression of vascular endothelial growth factor‐A (VEGFA) in colorectal cancer and promotes angiogenesis.^335^ In contrast, NF‐κB interacting long noncoding RNA (NKILA) was found to inhibit the IL‐6 production and angiogenesis through NF‐κB.^336^ NF‐κB in cancer cells promotes EMT.^337^ The expression of major EMT molecules (including Twist, Zinc finger transcription factor Snail2 (Slug) and Smad‐interacting protein1) is NF‐κB‐dependent, which initiate EMT and identify malignant phenotypes by enhancing the stemness and migration of cancer cells.^338^, ^339^ Additionally, NF‐κB activation also promotes EMT through other mechanisms, such as the matrix‐degrading enzyme.^340^ MMPs induced by NF‐κB promote the release of TGF‐β, EMT process, and hypoxia that contribute to metastatic dissemination.^341^, ^342^, ^343^ In addition, NF‐κB and cytokines (such as VEGF and TGF‐β) can directly stimulate the expression of metastasis‐related genes and promote tumor metastasis, such as exercise‐inducing factors and chemokines.^344^, ^345^ Inflammation and NF‐κB can also directly stimulate metastatic dissemination through EMT, increase the extravasation of cancer cells into the blood and lymphatic vessels, and prevent the death of connective tissue tumor cells.^346^ Taking liver cancer as an example, the functions of NF‐κB in tumorigenesis are schematically shown in Figure 3.

### 4.4 NF‐κB and cancer stem cells (CSCs)

CSCs are malignant cells that have the ability to self‐renew and differentiate into highly differentiated malignant cells. CSCs are the major reason for cancer recurrence, metastasis, and treatment resistance.^347^, ^348^ CSCs were first identified in leukemia and then isolated as CD34^+^CD38^–^ cells in the 1990s.^349^, ^350^ The following studies indicate that in other hematologic malignancies and solid tumors, CSCs express other different surface markers (such as CD133, nestin, and CD44).^351^, ^352^ The activities of cancer stem cells are controlled by many intracellular and extracellular factors and various signaling pathways. The functions of NF‐κB pathway in CSCs are complicated. NF‐κB is constitutively activated in various CSCs (including leukemia, glioblastoma, prostate, ovary, breast, pancreatic, and colon cancer), which mediates inflammation, cell proliferation, survival, maintenance, and expansion.^353^ NF‐κB could work alone or synergize with other signaling pathways to induce and promote the self‐renewal, proliferation, and metastasis of CSCs by mediating the expression of stem‐cell‐related transcriptional factors and genes (such as *Nanog*, *Sox2*, *Olig2*, *CD* *44*, *NKX3.1* and Krüppel‐like factor 4 (*KLF4*)).^354^ In breast cancer, NF‐κB activation causes CSC proliferation by activating Notch signaling in a cell‐involuntary way.^355^ In addition, high levels of NIK also induce the activation of the noncanonical NF‐κB pathway to regulate the self‐renewal and metastasis of breast CSCs.^356^ Therefore, drugs targeting NF‐κB pathway could be used to inhibit the proliferation and metastasis of cancer stem cells. The study has shown that in breast cancer stem cells, the anti‐alcoholism drug disulfiram inhibits TGF‐β through the extracellular regulated protein kinases(ERK)/NF‐κB/Snail pathway to induce tumor metastasis.^357^ Sulforaphane also inhibits the self‐renewal of triple‐negative breast cancer stem cells by inhibiting NF‐κB p65 subunit translocation and down‐regulating p52 and its transcriptional activity.^358^ The canonical NF‐κB pathway enhances the expression of the transcription factor Sry‐related HMG box 9 (*Sox9*), which is associated with enhanced pancreatic CSCs and invasiveness.^359^ In ovarian cancer, CD44^+^ CSCs enhance self‐renewal and metastasis by upregulating the expression of *RelA*, *RelB*, and *IKKα* and enhancing the activation of p50/RelA dimer.^360^ In colorectal cancer, the inflammatory mediator prostaglandin E2 activates NF‐κB through prostaglandin E Receptor 4‐phosphoinositide 3‐kinase to promote the formation, maintenance, and metastasis of CSCs.^361^ The transcription factor Foxp3 also interacts with NF‐κB, to inhibit the expression of the NF‐κB target gene *COX2*, and affect the self‐renewal and metastasis of colorectal CSCs.^362^

### 4.5 NF‐κB and tumor microenvironment

Tumorigenesis’ progress and metastasis rely on the environment where the tumor cells are located. The tumor microenvironment is composed of tumor cells, a variety of stromal cells, and immune cells, together with cytokines, chemokine, and other mediators, especially endothelial cells, cancer‐associated fibroblasts (CAFs), myeloid‐derived suppressor cells (MDSCs), and other numerous immune cells are critical for the tumor microenvironment.^363^, ^364^, ^365^ NF‐κB functions as the key regulator in many types of cells to shape the tumor microenvironment.

Cytokines produced by stromal cells, cancer cells, and immune cells are the key soluble factors for tumorigenesis, metastasis, and inflammation.^330^ Tumorigenesis is associated with tumor‐promoting cytokines, so it is important to understand the correlation between NF‐κB and pro‐tumorigenic cytokines. Tumor necrosis factor α (TNFα) is also one of the well‐studied tumor‐promoting cytokines, and its expression is enhanced in many cancers, which often indicates a poor prognosis.^302^, ^366^, ^367^ TNFα is mainly produced by activated neutrophils and macrophages, which induces other proinflammatory cytokines, including IL‐6 and IL‐1β, and accelerates tumorigenesis.^302^ Similar to TNFα, IL‐6 is another important and abundant proinflammatory cytokine in the tumor microenvironment. The functions of this NF‐κB‐dependent cytokine on inflammation and tumorigenesis have been well studied.^229^, ^367^, ^368^ IL‐6, mainly produced by malignant tumor cells and activated immune cells, initiates cancer‐related inflammation and stem cell expansion in an autocrine manner.^367^, ^369^ In addition, IL‐1α/β are also key proinflammatory cytokines produced by cancer cells and immune cells, which are NF‐κB‐dependent and promote the activation of NF‐κB and MAPK pathways and tumorigenesis.^370^, ^371^ IL‐17A activates NF‐κB and MAPK signaling pathways, thereby promoting tumorigenesis.^372^, ^373^, ^374^ The other IL‐1 family cytokine, IL‐33 is mainly expressed by epithelial cells, fibroblasts, and tumor cells, with a crucial function in allergy, autoimmunity, inflammation, and cancer.^375^, ^376^ Another important cytokine is TGF‐β, which is produced by a variety of cells, including fibroblasts, myeloid cells, T cells, and cancer cells. TGF‐β is essential for the differentiation of Treg and TH17 cells, making it a powerful inducer of tumor invasion and metastasis.^330^, ^343^

NF‐κB drives chemokine expression in tumor cells, stromal cells, and immune cells in the tumor microenvironment, especially CAFs.^377^, ^378^, ^379^ The activation of NF‐κB and other transcription factors (such as AP1 and STAT3) induces the expression of chemokines, which recruit more immune cells and further aggravate the inflammatory response.^380^, ^381^ Meanwhile, chemokines are important to stimulate the growth and metastasis of primary tumors, synthesizing growth factors, and angiogenesis. Although the functions of chemokines are complicated and uncertain in tumorigenesis and metastasis, chemokines still are important targets for cancer immunotherapy.^364^, ^382^, ^383^ For example, chemokine (C‐C motif) ligand 5 (CCL5) induces the formation of eukaryotic initiation factor 4F translation initiation complex in an mechanistic target of rapamycin (mTOR)‐dependent way, which mediates the quick upregulation of cyclin D1, c‐Myc, and defender against cell death‐1 (Dad‐1) protein expression to promote tumor cell proliferation in breast cancer.^384^ In addition, in human pancreatic cancer and mouse pancreatic tumor models (Pan02), the CCL5 level on tumor cells is elevated, while CD4^+^Foxp3^–^ effector T cells preferentially express C‐C chemokine receptor 5 (CCR5). When the CCR5/CCL5 signal is disturbed, either by inhibiting CCL5 expression on tumor cells or systemically administrating CCR5 inhibitors, the migration of Treg cells to the tumor microenvironment is reduced, which indicates that the chemokine CCL5 is required for Treg cells migration.^385^ Tumor‐related cytokines are mainly expressed by inflammatory cells, such as tumor‐associated macrophages (TAMs) and neutrophils. NF‐κB is mainly activated in these cells to induce the expression of cytokines (TNFα, IL‐1β, IL‐6, etc.), thereby promoting tumor survival and proliferation.^16^, ^323^ As the most abundant immune cells in the microenvironment, macrophages produce inflammatory cytokines and chemokines and produce proteases such as cysteine cathepsins, involved in activating cytokines to promote tumor development.^330^, ^365^ M1 macrophages activated by IFN‐γ and LPS can produce proinflammatory cytokines, chemokines, and enzymes to promote inflammation. In contrast, M2 macrophages induced by IL‐4, IL‐10, and IL‐13 can inhibit inflammation by releasing anti‐inflammatory mediators.^92^, ^386^, ^387^ M1 macrophages promote inflammation, while M2 macrophages create an immunosuppressive tumor microenvironment. The increase in p50 activity determines the M1 to M2‐ polarization of macrophages.^387^, ^388^, ^389^ Inhibiting NF‐κB transforms M2 macrophages to M1 macrophages because the polarization of M1 macrophages through IL‐1R and MyD88 into M2 macrophages requires IKKβ‐mediated activation of NF‐κB. Moreover, the impaired NF‐κB activation in TAMs also promotes the tumor‐killing activity of macrophages. When NF‐κB signaling is inhibited in TAMs, they become M1 phenotype with anti‐tumor cytotoxicity.^390^ NF‐κB could be a target for manipulating the phenotype of macrophages in the tumor microenvironment.

DCs are also important immune cells modulating anti‐tumor immunity in the tumor microenvironment. The canonical NF‐κB pathway in DCs can be inhibited by signals from the immune checkpoint molecule programmed cell death protein 1 (PD‐1), thereby inhibiting the production of cytokines.^391^ In contrast, the ligand of checkpoint like programmed cell death 1 ligand 1 (PD‐L1) is an NF‐κB target gene. The upregulation of PD‐L1 expression in cancer cells relies on NF‐κB activation triggered by many stimuli and activators, including oncogenes, stress, inflammatory cytokines, and chemotherapeutic drugs.^392^ In addition to macrophages and DCs, it has also been reported that IL‐1β induces NF‐κB activation in MDSCs to suppress the function of tumor microenvironment (TME), leading to tumor proliferation.^393^ In natural killer (NK) cells that directly kill tumor cells to exert anti‐tumor activity, the expression of cytotoxic effector molecules (such as perforin and granzyme B) ia also affected by NF‐κB.^394^, ^395^, ^396^ The activation of NF‐κB in NK cells can be stimulated by exogenous anti‐cancer drugs, like paclitaxel.^397^

As the important components of the tumor microenvironment, T cells and B cells in the tumor microenvironment could have tumor‐promoting or anti‐tumor functions.^398^ As mentioned above, the important stimuli of the NF‐κB pathway include TCR and BCR. The activation of conventional T cells requires the participation of the canonical NF‐κB pathway, which is required for CD8^+^ T cell proliferation and anti‐tumor immune response.^399^, ^400^ The development of Treg cells requires the participation of p65 and Rel. Rel is not only essential for the optimal steady‐state expansion of Treg cells in peripheral blood^150^ but also for the development of thymic Treg cells. p65 is crucial for Tregs maturation, and the maintenance of immune tolerance^401^ IKKβ‐dependent NF‐κB is activated in B cells that induce a cytokine LT, a heterotrimeric member of the TNF family that activates IKKα.^402^ The LT produced by B cells can activate IKKα in tumor cells. IKKα phosphorylates a site of the transcription factor E2F transcription factor 1 (E2F1) to promote E2F1 translocation and recruit to the target of the *Bmi1* gene. The IKKα–E2F1–*Bmi1* cascade activated by B cells controls prostate regeneration and tumor recurrence and regulates the renewal function of tumor stem cells.^403^

**FIGURE 4:**
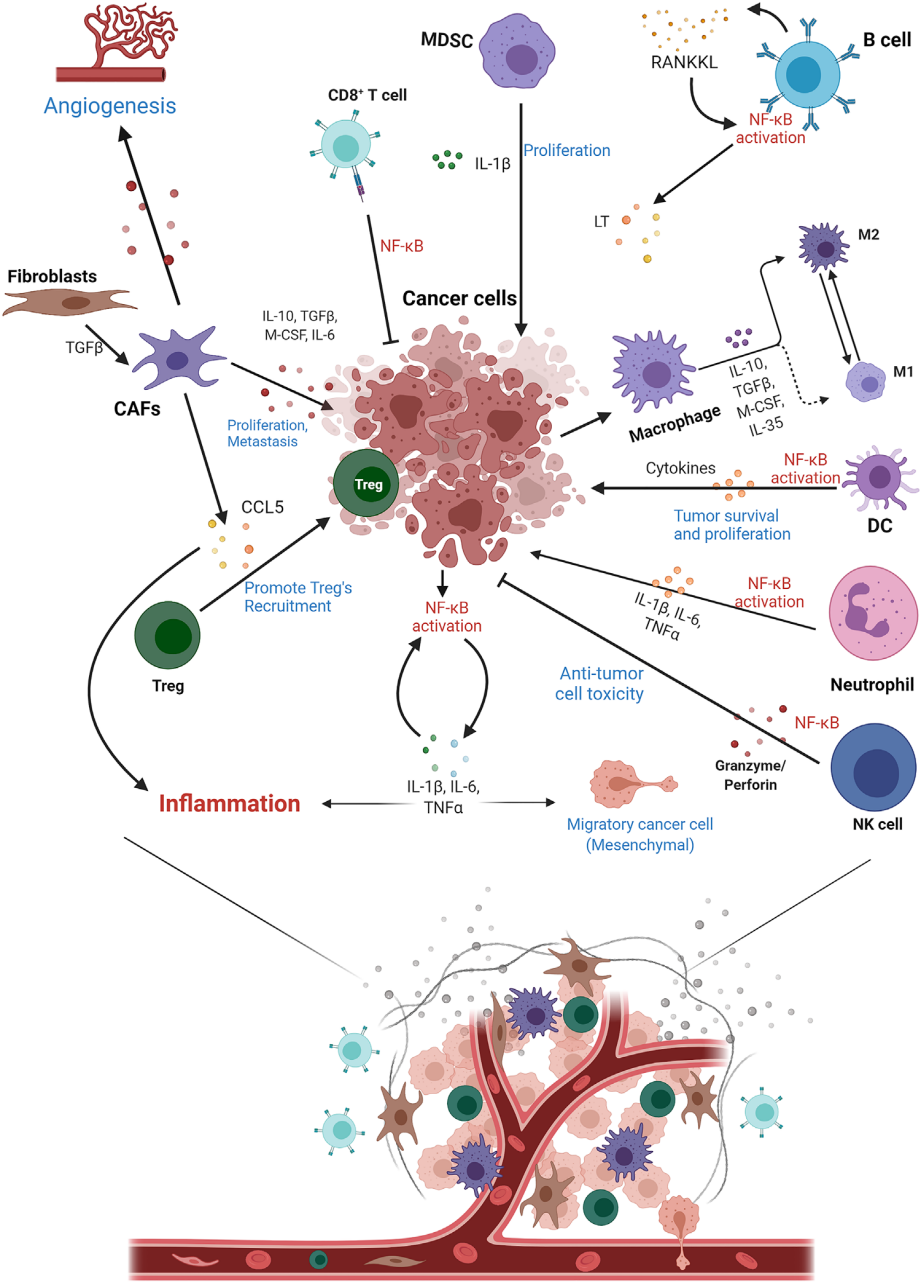
NF‐κB pathway modulates tumor microenvironment. The NF‐κB signaling pathway is tightly correlated to the various components of the tumor microenvironment. NF‐κB can not only drive the expression of chemokines in stromal cells, tumor cells, and immune cells in the tumor microenvironment but can also be activated in each cell to transcriptionally enhance the expression of cytokines and promote tumor proliferation and metastasis

The component that cannot be ignored in the tumor microenvironment is fibroblasts. Normal fibroblasts undergo changes in a self‐intrinsic way, and under the stimulation of tumor‐induced alterations tissue structure, TGF‐β, and hypoxia, they become tumor‐associated fibroblasts (CAFs) and express a large number of inflammatory factors and chemokines.^378^, ^379^ For example, IL‐1β is induced by the NF‐κB signaling pathway to promote inflammation, cancer cell proliferation, angiogenesis, and tumor metastasis.^404^ For example, in pancreatic ductal adenocarcinoma, CAFs mediate the exosomal metastasis and paracrine of tumor cells through producing cytokines (such as GM‐CSF and IL‐6), and this process requires the participation of NF‐κB. In breast cancer, locked nucleic acid (LNA)‐i‐miR‐221 inhibits miR‐221‐mediated NF‐κB activation to reduce the secretion of tumor‐promoting cytokines of CAFs.^405^ In brief, NF‐κB has complex functions on the different types of cells in the tumor microenvironment and might become a potentially effective therapeutic target. The function of the NF‐κB in the tumor microenvironment is shown in Figure 4.

### 4.6 NF‐κB and inflammation in cancer

The relationship between inflammation and tumor has become an important field of cancer research. It is well‐studied that inflammation is associated with autoimmune diseases, infection, and cancers.^5^ The well‐established examples of inflammation leading to cancer are HBV infection and hepatocellular carcinoma (HCC), and chronic *Helicobacter pylori* infection and mucous MALT lymphoma and gastric cancer.^25^, ^330^ NF‐κB is essential for the activation, differentiation, and effector functions of inflammatory T cells and innate immune cells and participate in the regulation of inflammasomes.^406^

**FIGURE 5:**
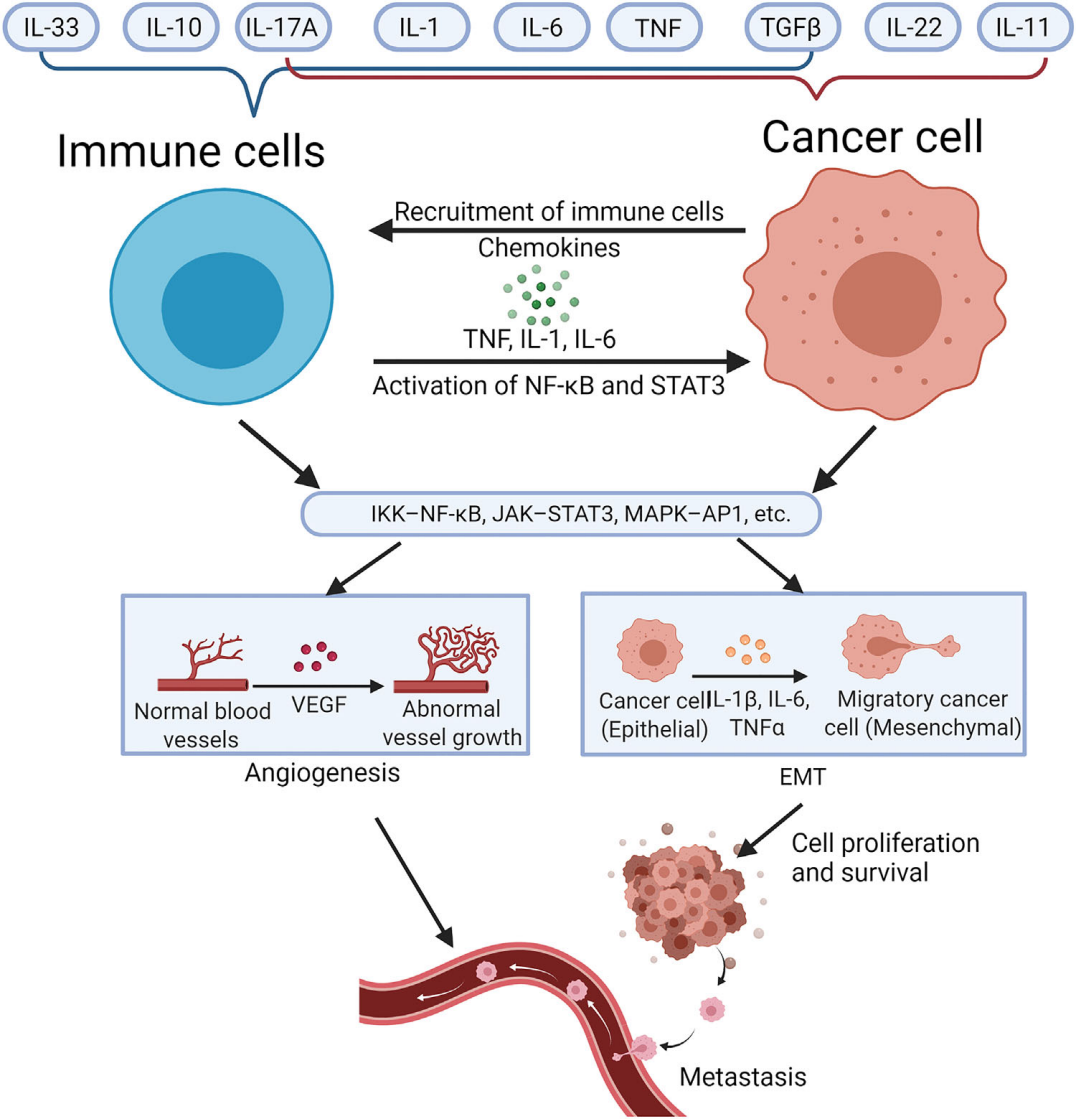
NF‐κB and inflammation in cancer. NF‐κB is involved in the interaction between immune cells and tumor cells. Cytokines such as TNFα, IL‐1, IL‐6, IL‐17A, and transforming growth factor‐β target both cancer cells and immune cells. IL‐1 and IL‐22 mostly target cancer cells, whereas IL‐10 and IL‐33 mainly act on immune cells. These cytokines activate the IκB kinase (IKK)‐NF‐κB, JAK‐signal transducer and activator of transcription 3 (STAT3), and MAPK‐AP1 signaling pathways of immune cells and cancer cells. The activation of NF‐κB in immune cells produces pro‐inflammatory cytokines, chemokines, and growth factors, such as TNFα, IL‐1, IL‐6, and vascular endothelial growth factor, thereby maintaining chronic inflammation and promoting angiogenesis. In cancer cells, pro‐inflammatory cytokines activate NF‐κB and STAT3 pathways, thereby stimulating cancer cell proliferation and survival, epithelial to mesenchymal transformation, invasion, angiogenesis, and metastasis

In addition to NF‐κB, the activation of other transcription factors such as AP1 and STAT3 can also induce the expression of chemokines, which recruit more immune cells (including T cells, B cells, macrophages, neutrophils, etc.) to aggravate the inflammatory response further.^380^, ^407^ As early as 2004, two studies showed the key roles of NF‐κB in inflammation‐driven colitis‐associated cancer and HCC.^229^, ^408^ These results indicate that tumorigenic pathogens cause chronic infection and inflammation, and chronic inflammation sequentially leads to malignant tumors by increasing cellular stress response and recruiting inflammatory immune cells.^5^, ^409^ However, chronic inflammation does not necessarily lead to cancer. As a chronic inflammatory disease, IBD is associated with colorectal cancer, but RA and psoriasis are chronic inflammatory diseases without obvious tumor outcomes.^410^ Another relevant evidence is that the loss of IKKβ in myeloid cells suppresses experimental colitis and colitis‐related cancers.^229^ Another area worthy of attention is tertiary lymphoid tissues. Clinical studies have shown that there are tertiary lymphoid tissues in the tumor microenvironment, and the presence of tertiary lymphoid tissues suggests a better prognosis.^411^, ^412^ The formation of tertiary lymphoid tissues is achieved by LTβR‐mediated noncanonical NF‐κB activation pathway to induce the expression of adhesion molecules and chemokines (like CCL19, CCL21, CXCL12, and CXCL13).^413^, ^414^ In general, the relationship between the NF‐κB signaling pathway and inflammation is profound and complex, and the precise mechanisms of NF‐κB, inflammation, and cancer in the current context need to be further studied. The functions of NF‐κB in the cross‐talking between immune cells and tumor cells are shown in Figure 5.

### 4.7 The therapeutic application of NF‐κB in cancer

With emerging research on NF‐κB and tumors, it is generally believed that NF‐κB is a valuable target for human cancer treatment. Considering the tumor‐promoting effects of NF‐κB, the current therapeutic strategy is inhibiting the activation of NF‐κB. These inhibitors include microbial and viral proteins, microRNA, non‐coding RNA, antioxidants, engineered active peptides, and various natural products to downregulate the canonical and noncanonical NF‐κB pathways.^415^ Due to the complexity of upstream stimulators and downstream target genes of NF‐κB, more than 1000 inhibitors could block the NF‐κB signaling pathway.^416^ Most of these inhibitors are found to have certain anti‐tumor effects using tumor cell models or animal models; their effects on human cancer treatment are unknown. Efforts have been made to develop specific and effective inhibitors with few side effects. A good example is bortezomib, a proteasome inhibitor, which has shown the inhibitory effect in MM.^417^ As mentioned earlier, NF‐κB is continuously activated in myeloma. Therefore, as a proteasome inhibitor, bortezomib was conceived to inhibit the degradation of ubiquitinated IκB, allowing IκB to bind to NF‐κB dimers and stay in the cytoplasm to inhibit transcription factor activity. In addition to bortezomib, carfilzomib and ixazomib are also approved as proteasome inhibitors for MM treatment by inhibiting NF‐κB.^296^ Two glucocorticoids prednisone and dexamethasone are also approved for the clinical treatment of MM. The treatment mechanism is to interfere with the phosphorylation of RNA polymerase II required for NF‐κB‐dependent transcription initiation.^418^ Except for inhibiting critical components of the NF‐κB pathway, blocking its downstream target genes or upstream stimuli is also a practicable therapeutic strategy. For example, denosumab, a monoclonal antibody that inhibits RANK ligand, and immunomodulatory drugs thalidomide, lenalidomide, and pomalidomide, which inhibit TNFα and IL‐1β, are also approved for the treatment of MM.^419^, ^420^ Similarly, in some DLBCL subtypes that exhibit continuous NF‐κB activation, BTK inhibitor has also been shown to have therapeutic value as a key molecule for BCR to activate the NF‐κB pathway.^421^ Other anti‐TNFα antibodies, including infliximab, adalimumab, and golimumab, have also been found to improve the therapeutic effect in breast cancer patients.^422^

Considering that these drugs do not directly target the core molecules of the NF‐κB pathway, but their upstream and downstream molecules, it is difficult to clarify how much the inhibitor functions on NF‐κB per se. Except for MM and some DLBCL subtypes, the strategy of directly inhibiting NF‐κB seems unsuccessful in other cancers. The success of the drugs mentioned above in MM and DLBCL cannot be replicated in other tumor treatments, suggesting NF‐κB has universal and complicated functions that could not be a fixed therapeutic target like PD‐1/PD‐L. Inhibition of NF‐κB may enhance the cancer treatment response to most traditional treatments and treatments developed in recent years such as immune checkpoint inhibitors; however, long‐term inhibition of the NF‐κB pathway perhaps causes serious side effects, especially impaired immune response and compensated anti‐tumor immune function.^423^ Given that NF‐κB regulates many physiological functions, the balance between the anti‐cancer effect and the side effects that impair normal physiological functions should be considered. Moreover, under drug‐mediated pressure, NF‐κB might cooperate with other signaling pathways to regulate pro‐survival genes (such as cyclin D1 and Bcl‐2) and upregulate the transcription of the genes, which helps tumor cells to develop drug resistance. Therefore, inhibiting NF‐κB alone without affecting other signaling pathways seems to be an unachievable presence.

One alternative strategy is to combine NF‐κB inhibitors with other treatments (such as chemotherapy and radiotherapy). For example, pentoxifylline (an REL inhibitor) combined with PD‐1 immunotherapy enhances the therapeutic effect.^424^ The combined usage of multiple NF‐κB inhibitors is also one of the feasible strategies. The study has reported using multiple NF‐κB inhibitors in combination and found that it improves the clinical treatment effect in MM.^425^ At present, there are many studies on NF‐κB inhibitors, which will not be summarized and discussed in this review, but most of the studies are conducted on in vitro cell models and animal models, and their value of human cancer treatment warrants further investigation. Recent clinical trials of cancer treatment targeting the NF‐κB signaling pathway are summarized in Table 2.

**TABLE 2:** Summary of part of recent NF‐κB pathway‐related inhibitor clinical trials

**Cancer type**	**Target**	**Main drug**	**Phase**	**NCT number**	Enrollment	Ref
MM	20S PI	Ixazomib	III	NCT01564537	722	^425^
KRAS G12D‐mutant/p53‐deficient NSCLC	26S PI	Bortezomib	II	NCT01833143	16	^448^
Classic Hodgkin lymphoma	26S PI	Bortezomib	II	NCT00967369	20	/
Head and neck cancer	26S PI	Bortezomib	I	NCT00011778	25	/
MM	SINE+26S PI	Selinexor + bortezomib + dexamethasone	II	NCT02343042	42	^449^
Head and neck adenoid cystic carcinoma	26S PI	Bortezomib	II	NCT00077428	25	^450^
Advanced oesophagogastric adenocarcinoma	26S PI	Bortezomib	I	/	18	^451^
Advanced gastric adenocarcinoma	26S PI	Bortezomib	II	/	16	^452^
Endocrine‐resistant metastatic breast cancer	26S PI	Bortezomib	II	/	9	^453^
Locally recurrent or metastatic squamous cell carcinoma of the head and neck	26S PI	Bortezomib	II	/	61	^454^
Malignant gliomas	26S PI + DNA alkylating agents	Bortezomib + Temozolomide	II	/	10	^455^
Plasma cell myeloma	20S PI	Ixazomib	II	NCT02765854	90	/
Waldenström's macroglobulinemia	20S PI	Ixazomib	II	NCT02400437	26	^456^
Waldenström's macroglobulinemia	20S PI+BTK	Carfilzomib + ibrutinib	III	NCT04263480	184	/
Advanced solid tumors	Na‐K ATPase	PBI‐05204	I	/	46	^457^
CLL	BTK	Ibrutinib	II	NCT01500733	86	^458^
Early‐stage chronic lymphocytic leukemia	NF‐κB inhibitor	Omega‐3 fatty acids	II	NCT00899353	16	^459^
Advanced solid tumors and lymphomas	IKK inhibitor	Bardoxolone methyl	I	/	44	^460^
Primary CNS lymphoma	BTK	Ibrutinib	I	NCT02315326	13	^461^
Waldenström's macroglobulinemia	BTK	BGB‐3111	III	NCT03053440	229	/
NSCLC	RANKL	Denosumab	III	NCT02129699	509	^462^
Giant cell tumor of bone	RANKL	Denosumab	III	NCT03259152	30	/
Urothelial carcinoma	RANKL	Denosumab	II	NCT03520231	50	/
Melanoma	RANKL	Denosumab	I	NCT03161756	72	/
MM	BCL‐2	Venetoclax	I/II	NCT01794520	51	^463^
MM	IL‐1R	Anakinra	I	NCT02492750	14	/
ALL	Phosphodiesterase	Pentoxifylline	III	NCT02451774	44	/
Triple negative breast cancer	CD40	CDX‐1140	I	NCT05029999	45	/
Metastatic melanoma	CD40	APX005M	I	NCT03597282	22	/
NSCLC	CD40	APX005M	I	NCT03123783	400	/
Soft tissue sarcoma	CD40	APX005M	II	NCT03719430	27	
Metastatic pancreatic adenocarcinoma	CD40	APX005M	I	NCT03214250	129	^464^
Melanoma	CD40	SEA‐CD40	II	NCT04993677	200	/
NSCLC	CD40	SEA‐CD40	I	NCT02376699	159	/
Melanoma	CD40	CP‐870,893	I	NCT01103635	25	/
DLBCL	CD40	SGN‐40	II	NCT00529503	151	/
Non‐Hodgkin lymphoma, DLBCL	BAFF‐R	VAY736	I	NCT04903197	86	/

Abbreviations: ALL, acute lymphoblastic leukemia; BTK, Bruton's tyrosine kinase; CLL, chronic lymphocytic leukemia; diffuse large B‐cell lymphoma. All NCT numbers were obtained from https://clinicaltrials.gov/; IKK, IkappaB kinases; MM, multiple myeloma; NSCLC, non‐small cell lung cancer; PI, proteasome inhibitor; RANKL, receptor activator of NF‐κB ligand; SINE, selective inhibitor of nuclear export.

## 5 CONCLUSION

Since NF‐κB was discovered in 1984, extraordinary efforts have been being made to understand the functions and regulating mechanisms of NF‐κB for 35 years, leading to significant progress. There has been convincing evidence to support the connection between NF‐κB, cancer, and inflammation, and it has been accepted that the NF‐κB pathway plays a key role in immune homeostasis, chronic inflammation, tumorigenesis, and development. Based on the numerous upstream stimuli and downstream target genes of NF‐κB currently known, we conclude that NF‐κB has a wide range of functions, like a hub with a huge communication network. Nevertheless, there are still many unknowns about the comprehensive mechanisms and functions of NF‐κB that we need to further explore. Recently, more and more drugs targeting NF‐κB have been developed and examined. It should be noted that considering the diversity of upstream stimulus and downstream target genes of NF‐κB, to complete inhibition of NF‐κB as a therapeutic method might not be a practicable strategy. Future investigation on NF‐κB as an anti‐cancer target should focus on inhibiting its tumor‐promoting effects caused by pathological NF‐κB activation while avoiding affecting their normal physiological functions; meanwhile, the combination of multiple drugs targeting different key regulators of the NF‐κB pathway, even multiple pathways, is also attractive.

## CONFLICT OF INTEREST

The authors declare no conflict of interest.

## ETHICS STATEMENT

The authors declare that ethics approval was not needed for this study.

## AUTHOR CONTRIBUTIONS

T.Z., H.Z., and H.H. conceived, wrote, and edited the manuscript; C.M. and Z.Z. provided significant assistance.

## Data Availability

Not applicable.
